# MS Binding Assays for Glycine Transporter 2 (GlyT2) Employing Org25543 as Reporter Ligand

**DOI:** 10.1002/cmdc.202000342

**Published:** 2020-09-10

**Authors:** Thomas M. Ackermann, Lars Allmendinger, Georg Höfner, Klaus T. Wanner

**Affiliations:** ^1^ Department of Pharmacy, Center for Drug Research Ludwig-Maximilians-Universität Munich Butenandtstraße 7 81377 Munich Germany

**Keywords:** binding assay, glycine transporter 2, liquid chromatography, mass spectrometry, neurotransmitter

## Abstract

This study describes the first binding assay for glycine transporter 2 (GlyT2) following the concept of MS Binding Assays. The selective GlyT2 inhibitor Org25543 was employed as a reporter ligand and it was quantified with a highly sensitive and rapid LC‐ESI‐MS/MS method. Binding of Org25543 at GlyT2 was characterized in kinetic and saturation experiments with an off‐rate of 7.07×10^−3^ s^−1^, an on‐rate of 1.01×10^6^ M^−1^ s^−1^, and an equilibrium dissociation constant of 7.45 nM. Furthermore, the inhibitory constants of 19 GlyT ligands were determined in competition experiments. The validity of the GlyT2 affinities determined with the binding assay was examined by a comparison with published inhibitory potencies from various functional assays. With the capability for affinity determination towards GlyT2 the developed MS Binding Assays provide the first tool for affinity profiling of potential ligands and it represents a valuable new alternative to functional assays addressing GlyT2.

## Graphical abstract

**Assay assayed**. MS Binding Assays enabled us to characterize the binding of the reporter ligand Org25543 towards the glycine transporter GlyT2 in kinetic and saturation as well as the binding of test compounds in competition experiments. Comparing the results with those from literature showed that this GlyT2 MS Binding Assay is a viable alternative to functional assays.

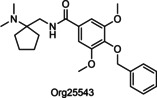

## 1 Introduction

Today the treatment of chronic pain is a widespread problem in pain therapy. Patients who are suffering from chronic pain do not just have to deal with normal pain sensation but also with hyperalgesia (amplified pain sensation to a noxious stimulus) and/or allodynia (pain sensation after an innocuous stimulus like a light touch) especially when the reason for pain is a nerve injury in the peripheral or central nervous system (neuropathic pain).^[1]^ Furthermore, this can lead to a worse quality of life due to physical, psychological and social problems like decreased physical activity, sleep disturbances, anxiety, depression, decreased social interactions with family and friends and inability to work.^[2]^ Currently, patients with chronic pain are treated with the typical analgesics like opioids or nonsteroidal anti‐inflammatory drugs or with anticonvulsants or tricyclic antidepressants (especially for neuropathic pain) but these therapies are often not very satisfying as the pain relief effect is to low and the drugs are associated with unwanted side effects.^[1]^ Thus there is a strong need for new strategies for the treatment of chronic pain.

**Figure 1:**
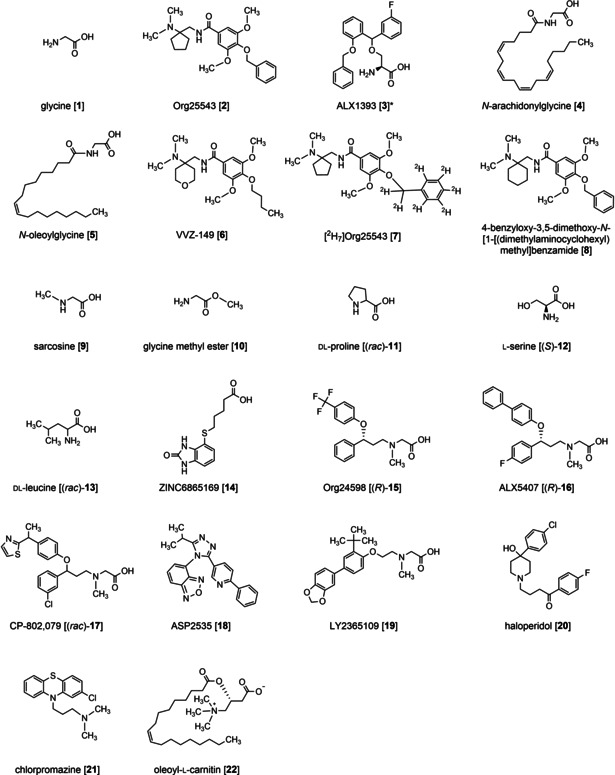
Structures of Org25543, used as a GlyT2 marker, [^2^H_7_]Org25543, used as internal standard, and GlyT ligands. * Stereochemistry not unequivocally characterized either by literature or by manufacturer or vendor.

The dorsal horn of the spinal cord acts as a control center for the transmission of painful information between periphery and brain. Part of this control center are glycinergic neurons with glycine (Figure 1; **1**) as inhibitory neurotransmitter. These glycinergic neurons can inhibit the transmission of incoming information from the periphery by activating glycine receptors (GlyR) on the postsynaptic neuron due to glycine binding which triggers a Cl^−^ influx and leads to a hyperpolarization. This mechanism was installed from nature as control mechanism to distinguish between noxious and innoxious information.^[1]^ A decreased function of this inhibitory mechanism, for example after inhibition of glycine receptors with strychnine or nerve injury, leads to a loss of this ability which results in hyperalgesia and allodynia.^[3]^ Thus an increased inhibitory glycinergic neurotransmission could reduce the symptoms of these diseases. One strategy to increase the inhibitory neurotransmission is to inhibit the reuptake of glycine in presynaptic neurons and glial cells mediated by the corresponding neurotransmitter transporters, that is, glycine transporter 1 (GlyT1) and glycine transporter 2 (GlyT2).^[4]^ Although the inhibition of GlyT1 is showing antinociceptive effects[^5^, ^6^, ^7^] this work will just concentrate on GlyT2. GlyT2 is a member of the Na^+^/Cl^−^‐dependent solute carrier 6 (SLC6) family and occurs mainly at the presynaptic terminal of inhibitory glycinergic synapses. There it has two main functions: First, in a co‐transport with Na^+^ and Cl^−^ (stoichiometry: 3 Na^+^+1 Cl^−^+1 glycine) glycine is removed from the synaptic cleft of inhibitory glycinergic synapses into the presynaptic neuron to terminate the glycinergic neurotransmission. This leads to a low‐nanomolar concentration of glycine in the synaptic cleft, which is insufficient for a significant GlyR activation. Second, GlyT2 has a recycling function for glycine. Due to the transport back into the presynaptic neuron the glycine concentration increases there to ∼10–20 mM which is then high enough to refill synaptic vesicles via the vesicular inhibitory amino acid transporter (VIAAT) with glycine whereupon it can be released again into the synaptic cleft. So, the glycinergic neurotransmission can be upheld.^[1]^

**Figure 2:**
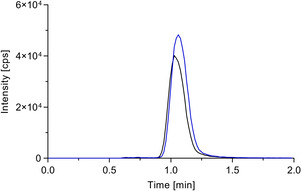
MRM chromatogram of a matrix standard containing 100 pM Org25543 (*m*/*z* 413.2/368.3, blue) and 100 pM [^2^H_7_]Org25543 (*m*/*z* 420.3/375.4, black). For LC a Luna 3 μ C8(2) (50 mm×2 mm, 3 μm) column was used as stationary phase in combination with a mobile phase consisting of ammonium bicarbonate buffer (5 mM, pH 7.8)/acetonitrile (20 : 80, *v*/*v*) at a flow rate of 600 μL min^−1^. In routine LC‐ESI‐MS/MS runs, the eluent was directed to waste from 0.0–0.6 min.

**Figure 3:**
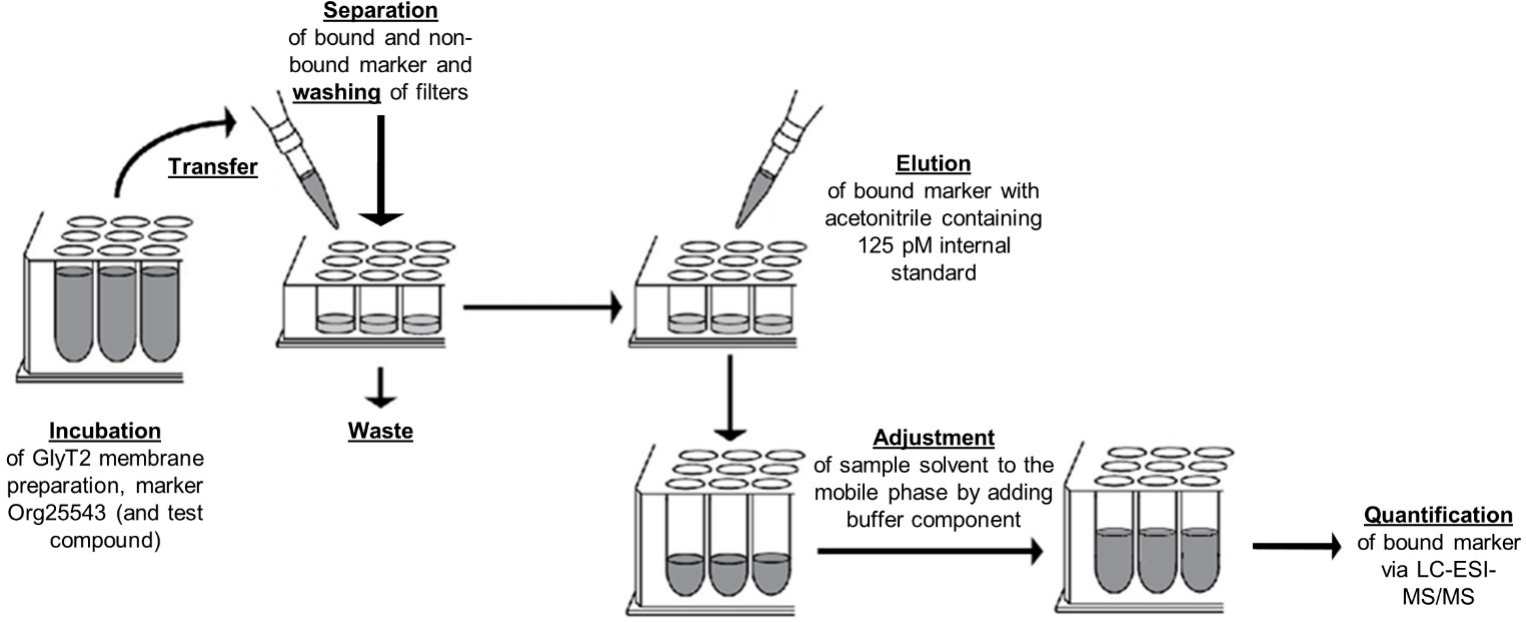
Workflow of the developed GlyT2 MS Binding Assays

**Figure 4:**
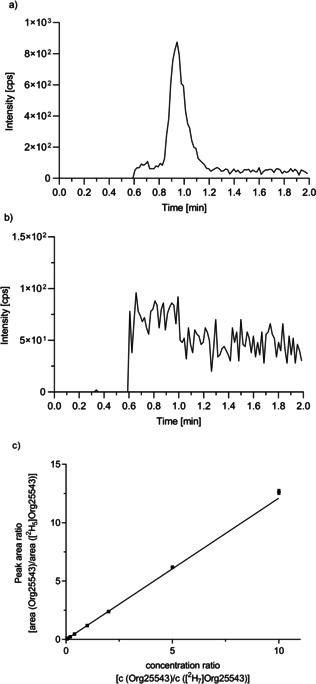
Validation of the developed LC‐ESI‐MS/MS method. a) MRM chromatogram for Org25543 at the LLOQ of 5 pM. b) MRM chromatogram for a matrix blank (for demonstration of selectivity). c) Representative calibration for Org25543 over a range from 5 pM to 1 nM employing [^2^H_7_]Org25543 (100 pM) as internal standard. The peak area ratio of marker and internal standard (mean±SD, *n*=3) was plotted against the concentration ratio of marker and internal standard. The corresponding calibration function obtained by linear regression was *y*=1.212*x*−0.008975; *R* ^2^=0,9990.

**Figure 5:**
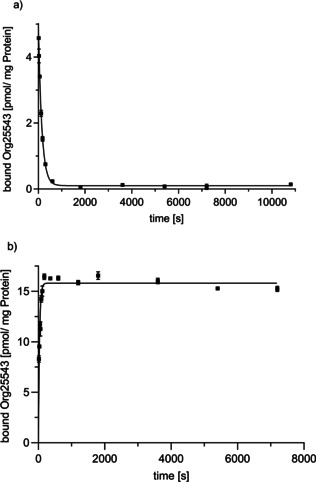
Representative kinetic experiments showing the time dependence of dissociation and association of Org25543 at GlyT2. a) Dissociation kinetics of the target–marker complex determined for Org25543 at GlyT2 after displacement by an excess of **8**. Specific binding (obtained after subtraction of nonspecific binding from total binding) plotted against time (mean±SD, *n*=3). b) Association kinetics determined for Org25543 at GlyT2. Specific binding (obtained after subtraction of nonspecific binding from total binding) was plotted against time (mean±SD, *n*=3).

**Figure 6:**
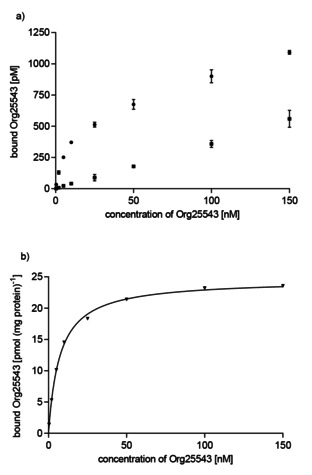
Representative saturation experiment showing total, nonspecific and specific binding of Org25543 at GlyT2. a) Experimental data (mean±SD, *n*=3) for total (▪) and nonspecific (•) binding. Nonspecific binding for nominal concentrations of Org25543 less than 2 nM was extrapolated after modeling of experimentally determined nonspecific binding at concentrations of Org25543≥2 nM by linear regression. b) Specific binding (▾; means, pmol (mg protein)^‐1^) calculated as the difference of total binding and nonspecific binding from a) and saturation isotherm generated by nonlinear regression.

By increasing glycine concentration in the synaptic cleft and thus glycinergic neurotransmission, GlyT2 inhibition can be expected to be active against pain, which could be demonstrated in animal models. Org25543 (Figure 1; **2**) and ALX1393 (Figure 1; **3**) represent the two so far best characterized GlyT2 inhibitors, which were shown to be able to reduce allodynia in animals due to their ability to inhibit the glycine transport into the presynaptic neuron.[^7^, ^8^, ^9^, ^10^] Unfortunately, both compounds exhibit considerable disadvantages. ALX1393 shows an insufficient availability in the CNS after intravenous injection (only 5 % cross the blood‐brain‐barrier)^[11]^ whereas a prolonged exposure of Org25543 to GlyT2 leads to a reduced glycinergic neurotransmission. The latter is presumably due to the reduction of glycine transport in the glycinergic neuron.^[12]^ When GlyT2 is completely blocked, the glycine concentration in the presynaptic neuron cannot reach the required concentration level to refill the synaptic vesicles with glycine and thus no new glycine can be released into the synaptic cleft, which results in a decreased glycinergic neurotransmission. Despite these disadvantages researchers are still looking for new GlyT2 inhibitors with better and safer pharmacological profiles. Right now clinical trials for the GlyT2 inhibitor VVZ‐149 (Figure 1; **6**) from Vivozone, which additionally acts as antagonist at the 5‐HT_2A_ and P2X3 receptors, for the treatment of postoperative pain are in progress.[^13^, ^14^, ^15^, ^16^] Furthermore, most recent research aims at the development of lipid‐based GlyT2 inhibitors like *N*‐arachidonylglycine (NAGly; Figure 1; **4**) or *N*‐oleoylglycine (NOGly; Figure 1; **5**),[^1^, ^17^, ^18^, ^19^, ^20^, ^21^] which show positive effects in neuropathic pain models but fewer side effects.

To find new inhibitors for GlyT2 efficient screening tools are necessary. Until now all methods (to the best of our knowledge), which have been developed for the screening of new inhibitors, are assays aiming for the identification of compounds with a biological activity at GlyT2 like radioisotope labeled glycine uptake assays,[^22^, ^23^, ^24^, ^25^, ^26^] electrophysiological assays[^11^, ^18^, ^19^, ^20^, ^21^] or fluorescent imaging plate reader (FLIPR) membrane potential assays.^[27]^ But there is no assay measuring the binding affinities of compounds towards GlyT2 so far. As the determination of binding affinities is in general (e. g., no living cells are needed) less elaborate than the performance of functional assays and binding affinities by being direct proportional to the Gibbs free energy (▵*G* ^0^) are required as basis for molecular modelling studies, we aimed at the development of a binding assay addressing GlyT2. To this end we intended to use the concept of MS Binding Assays, which was established in our group during the last years and was employed already for GlyT1 and several other target proteins.[^28^, ^29^, ^30^, ^31^, ^32^, ^33^, ^34^, ^35^, ^36^, ^37^, ^38^, ^39^] MS Binding Assays (as described in Section 2.3.) are performed similar to radioligand binding assays but instead of using a radioisotope labeled reporter ligand an unlabeled reporter ligand, which is also termed “native marker”, “MS Marker” or simply “marker”, is employed. The incubation step and the separation step of unbound marker and target‐marker complexes (either by filtration or centrifugation) are the same for both techniques, however, the quantification of the bound reporter ligand differs. In MS Binding Assays the bound marker is quantified typically after its liberation from the target‐marker‐complex with an organic solvent by LC‐MS, whereas in radioligand binding assays the target‐bound marker that remains on the filters after the separation step is quantified directly by liquid scintillation counting (LSC).

For the development of the desired MS Binding Assay a reporter ligand was needed, which shows an appropriate affinity to GlyT2 (preferably in the low‐nanomolar range) as well as a good capability for the quantification by LC‐ESI‐MS/MS (liquid chromatography coupled to an electrospray ionization interface and following quantification by tandem mass spectrometry), which commonly proves to be well suited for this purpose.^[28]^ Unfortunately, the number of commercially available GlyT2 inhibitors, which show a biological activity at GlyT2 in the low‐nanomolar range, is very low. To be more precise, only two compounds, that is, Org25543 and ALX1393 with reported IC_50_ values from 16 to 100 nM in functional assays, appeared suitable for this task.[^11^, ^23^] As no LC‐ESI‐MS/MS method has been described for either GlyT2 inhibitor so far, it was necessary to develop a method which allows a highly sensitive quantification of these compounds. Taking into account that the compounds have to be quantifiable down to about 0.01 *K*_d_, as a rule of thumb,[^29^, ^31^] the developed LC‐ESI‐MS/MS method should be able to quantify the reporter ligand in the low‐picomolar range. On the basis of the results obtained during the method development process, the compound that appears to be better suited for this task, that is, either Org25543 or ALX1393 by showing better LC‐MS properties, should be established as reporter ligand. For the chosen reporter ligand, the developed LC‐ESI‐MS/MS method should be validated regarding linearity, lower limit of quantification (LLOQ), accuracy, precision and selectivity. After that the kinetics (off‐ and on‐rates) as well as the affinity of the chosen reporter ligand at GlyT2 should be examined in dissociation, association and saturation experiments and, subsequently, known GlyT ligands should be characterized in competition experiments. In the end, the results of these competition experiments should be compared to those from functional GlyT2 assays from literature as far as such data for the used competitors are available for GlyT2. Although other data from neurotransmitter transporters show that the direct comparability of affinities and potencies from functional assays is limited, such a comparison should at least enable to estimate the plausibility of the determined binding affinities.

## 2 Results and Discussion

### 2.1 LC‐ESI‐MS/MS method

For the first step in the development of the desired MS Binding Assay a reliable LC‐ESI‐MS/MS method had to be established to which end a triple quadrupole mass spectrometer coupled to a pneumatically assisted electrospray ionization (ESI) source should be used. Thereby, the mass spectrometer should be operated in the multi‐reaction monitoring (MRM) mode to achieve the required selectivity and sensitivity to analyze binding samples. For ALX1393 (IC_50_ of 25–100 nM[^11^, ^40^]), one of the two possible reporter ligands, neither the [*M*+H]^+^ parent ion nor the fragment ions had been described for ESI‐MS/MS so far, whereas for Org25543 (IC_50_ of 16–31 nM[^23^, ^27^]) the [*M*+H]^+^ is known (*m*/*z* 413.3^[23]^) but the fragment ions are not. Our experiments revealed the expected [*M*+H]^+^ parent ions with *m*/*z* 396,1 for ALX1393 and *m*/*z* 413.2 for Org25543 as well as following fragment ions for ALX1393: *m*/*z* 291.3, 185.1, 184.8, 165.1, 91.1 and Org25543: *m*/*z* 368.3, 271.2, 243.2, 162.1, 134.1, 98.2, 91.2, 81.2, respectively (see Figure S1 in the Supporting Information). The mass transitions with the highest intensities (ALX1393→*m*/*z* 396.1/291.3 and Org25543→*m*/*z* 413.2/368.3) were selected for further method development.

Next, after ALX1393 and Org25543 had been characterized by ESI‐MS/MS and the compound‐dependent parameters of the mass spectrometer had been optimized, the LC method should be developed. For this purpose, we decided to use a method running under reversed‐phase (RP) conditions. RP‐HPLC does not just enable an easy and fast method development due to its simple retention principle but it also has the advantage to separate the analyte from matrix components effectively which is important to avoid matrix effects potentially disturbing analyte detection. According to our experience most of the matrix components generated during the assay procedure are highly polar and will thus by being hardly retained elute close to the void time. Due to this it was our aim to achieve a capacity factor (*k*) of the analyte between 1 and 2, which should be large enough to avoid significant matrix effects. Hence, several RP stationary phases and different mobile phases consisting of acetonitrile or methanol as organic component and an aqueous buffer made from volatile buffer salts like ammonium formate or ammonium bicarbonate in various ratios, were tested to identify conditions suitable for this purpose (data not shown). During this method development ALX1393 was found to exert much lower intensities compared to Org25543, its suitability for LC‐ESI‐MS/MS detection thus being worse than that of Org25543, but still high enough to guarantee a quantification of ALX1393 down to picomolar concentration levels (Figure S2). The following two concerns, however, clearly indicated, that ALX1393 was not an ideal candidate to be used as reporter ligand in binding assays. First, the stereochemistry of ALX1393, which is likely to have distinct influence on its binding affinity, is not defined for one of the two chiral centers. Therefore, commercially available ALX1393 can even not be expected to be just a single stereoisomer with unknown configuration at one chiral center but a mixture of diastereomers, possibly varying in their ratio when different batches from the same or even more from different vendors are used. Secondly, despite its described nanomolar potency in functional assays Table 1 we determined an affinity characterized by a *K*_i_ value of about 1 μM at GlyT2 in our competition experiments (see Section 2.7.). Compounds in this affinity range are poorly suited for filtration‐based binding assays, as a substantial loss of bound reporter ligand during the washing step after separation of bound from unbound reporter ligand is likely to occur. Therefore, we focused on Org25543 as reporter ligand addressing GlyT2, for which finally a LC method that meets the required needs, could be developed. It is based on a Luna C8(2) (50 mm×2 mm, 3 μm) column as well as 5 mM ammonium bicarbonate buffer (pH 7.8) and acetonitrile (20 : 80, *v*/*v*) as mobile phase leading to the desired retention behavior for Org25543, with the capacity factor (*k*) amounting to 1.69. The mentioned conditions enable to run the method under acceptable back pressures (∼120 bar) with a flow rate of 600 μL min^−1^ which makes chromatographic cycle times of only 2.0 min possible and guarantees therefore a considerable throughput.

**Table 1:** Affinities of known GlyT ligands determined in GlyT2 MS Binding Assays (mean±SEM, *n*=3–5) with a comparison of biological activities determined in [^3^H]glycine uptake or oocyte glycine current assays published in literature.

Compound	Affinity, p*K*_i_ (GlyT2 MS Binding Assays)	Biological activity, IC_50_ ([μM], literature)	pIC_50_ (literature; calcd)
glycine [**1**]	1.98±0.08	6–1801^[11,18,22,27,47–51][b]^	5.22–2.74
ALX1393 [**3**]	5.93±0.03	0.026–0.100[^11^, ^40^]	7.59–7.00
*N*‐arachidonylglycine [**4**]	5.40±0.09	5.1±3.1^[52]^	5.29
*N*‐oleoylglycine [**5**]	5.63±0.06	0.500^[19]^	6.30
4‐benzyloxy‐3,5‐dimethoxy‐*N*‐[1‐[(dimethylaminocyclohexyl) methyl]benzamide [**8**]	7.38±0.01	0.084±0.003^[23]^	7.08
sarcosine [**9**]	2.2 mM, 98±6 %^[a]^ (<3.03)	>1000^[22]^	<3
glycine methyl ester [**10**]	1.11±0.06	84±5 %^[47],[c]^	
dl‐proline [(*rac*)‐**11**]	1.50±0.11	95±5 %^[47],[c]^	
l‐serine [(*S*)‐**12**]	180 mM, 97±4 %^[a]^ (<1.11)	89±7 %^[47],[c]^	
dl‐leucine [(*rac*)‐**13**]	10 mM, 99±3 %^[a]^ (<2.37)		
ZINC6865169 [**14**]	600 μM, 77±6 %^[a]^ (<3.59)	0.518±0.066^[40]^	6.29
Org24598 [(*R*)‐**15**]	300 μM, 101±5 %^[a]^ (<3.89)	>100^[53]^	<4
ALX5407 [(*R*)‐**16**]	600 μM, 90±5 %^[a]^ (<3.59)	>100^[54]^/1.8^[40]^	<4/5.74
CP‐802,079 [(*rac*)‐**17**]	4.64±0.001	>10^[55]^	<5
ASP2535 [**18**]	5.99±0.11	4.6±0.290^[56]^	5.34
LY2365109 [**19**]	10 μM, 94±10 %^[a]^ (<5.37)	>30^[57]^	<4.52
haloperidol [**20**]	4.90±0.06	13±2^[58]^	4.89
chlorpromazine [**21**]	4.55±0.01	21±4^[58]^	4.68
oleoyl‐l‐carnitine [**22**]	5.58±0.06	0.340^[18]^	6.47

[a] remaining Org25543 binding (% of specific binding) at the highest compound concentration [b] *K*_m_ and EC_50_ values. [c] Glycine uptake in presence of 1 mM competitor (% of control).

**Figure 7:**
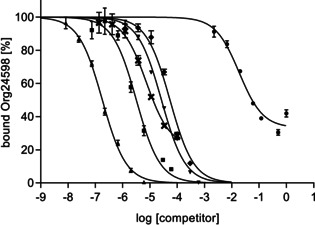
Representative competition curves obtained in GlyT2 MS Binding Assays for **8** (▴), ALX1393 (▪), glycine (•), CP‐802,079 (♦), haloperidol (▾) and *N*‐oleoylglycine (**x**). The experimental data represent specific binding (mean±SD, *n*=3) of Org25543 (10 nM) at various concentrations of the competitors. 100 % binding was equivalent to specific binding of Org25543 without any competitor and 0 % to nonspecific binding. For compounds, which are exhibiting incomplete inhibition, the bottom level was not constrained to the nonspecific binding level (0 %).

Finally, to improve the robustness of the quantification method being developed for Org25543 an internal standard should be employed.^[28]^ As such the seven times deuterated form of Org25543 (Figure 1; **7**) should be used, which was synthesized in‐house (see Section 2.2). Still, as already discussed by us,^[29]^ it is not mandatory to use an isotopically labeled form of the reporter ligand as internal standard. Any other compound which coelutes with the marker and which can be well quantified via LC‐ESI‐MS/MS under the same conditions as the analyte is in principle appropriate for this use, too.

A representative chromatogram of a matrix standard containing 100 pM Org25543 and 100 pM [^2^H_7_]Org25543, which was acquired under the above described LC‐ESI‐MS/MS conditions, is depicted in Figure 2. As can be seen from it the signal intensity for Org25543 for this low concentration is still high, the retention time is short and analyte and internal standard co‐elute, the method thus fulfilling the conditions required for a sufficiently sensitive and robust quantification of the reporter ligand and a reasonable throughput.

### 2.2 Synthesis of the internal standard [^2^H_7_]Org25543

**Scheme 1:**
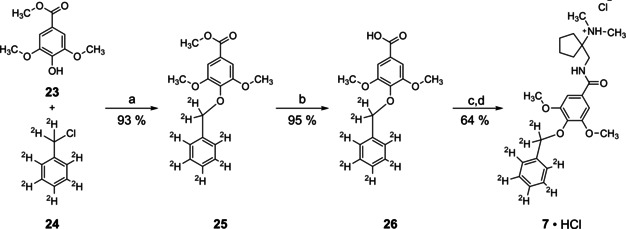
Synthesis of [^2^H_7_]Org25543. a) K_2_CO_3_, KI, DMF, 80 °C, 24 h; b) LiOH, THF/H_2_O, RT, 68 h; c) 1‐(aminomethyl)‐*N*,*N*‐dimethylcyclopentane‐1‐amine,^[41]^ HOBt, EDCI, NEt_3_, CH_2_Cl_2_, RT, 18 h; d) 4 M HCl, dioxane, 0 °C.

For the synthesis of [^2^H_7_]Org25543 (**7**; Scheme 1) we started from methyl syringate [**23**] and [^7^H_2_]benzyl chloride [**24**], both of which are commercially available. By subjecting them to a Williamson ether synthesis, ether **25** was obtained in 93 % yield. Hydrolysis of the ester function of **25** under mild basic conditions with LiOH furnished acid **26** (yield: 95 %). Subsequent reaction of carboxylic acid **26** with 1‐(aminomethyl)‐*N*,*N*‐dimethylcyclopentane‐1‐amine after prior activation with 3‐(ethyliminomethyleneamino)‐*N*,*N*‐dimethylpropan‐1‐amine (EDCI) and benzotriazole‐1‐ol (HOBt) in the presence of NEt_3_ (in CH_2_Cl_2_, RT, 18 h), delivered the desired deuterated analogue [^2^H_7_]Org25543 [**7**] in 70 % yield. Upon treatment of the free base **7** with HCl (4 M) in dioxane the hydrochloride **7**⋅HCl was obtained (yield 91 %, 64 % over 2 steps).

### 2.3 Preliminary experiments and considerations for the development of GlyT2 MS Binding Assays regarding its general setup and conditions

So far, no binding assay for GlyT2 has been described in literature and thus no data as initial guide for experimental conditions exist. Therefore, the experimental conditions established for the GlyT1 MS Binding Assay should serve as such.^[29]^ As 10 mM HEPES, 120 mM NaCl, 2 mM KCl, 1 mM MgCl_2_, 1 mM CaCl_2_ with pH 7.5 proved to be suitable for GlyT1 binding experiments, we reasoned that the same should be true for GlyT2. Vacuum filtration over glass fiber filters should be used after incubation, to separate target‐bound‐marker from unbound marker. To remove residual unbound marker in the filters after the separation step, they later have to be washed several times, for which purpose, a 154 mM ammonium acetate washing buffer (pH 7.4) should be used here for GlyT2 as for GlyT1 before.^[29]^ As buffer components from the washing procedure will remain on the filters and elute from the filters during the elution of the marker, they will contribute to the final sample composition. Ammonium acetate buffer has been found to be favorable for ESI‐MS/MS measurements due to its volatile character. Another important issue concerning the quality of the results of the binding assay is related to nonspecific binding of the MS marker to the glass fiber filters. This should be limited to a minimum by means of a pretreatment with suitable compounds. Since such compounds contribute to the final sample composition and matrix effects as well, suitable filter pretreatment reagents and conditions had to be evaluated. Preliminary experiments revealed that Org25543 shows very high filter binding, in case filters are not pretreated. Thus, several pretreatment agents described by Scott et al.^[42]^ were tested from which an aqueous 0.5 % (*m*/*m*) polyethylenimine (PEI) and an aqueous 1 % (*m*/*m*) Tween20 solution exhibited best and almost similar results in reducing the filter binding of Org25543 (for details, see Figure S3). Finally, 0.5 % PEI was selected as pretreatment agent for the reduction of filter binding, since a higher matrix effect was observed when filters were pretreated with Tween20 compared to the ones pretreated with PEI. In the last step of the binding experiment, the target‐bound‐marker remaining on the filters has to be liberated and eluted. In former MS Binding Assays the solvents acetonitrile and methanol have already been shown to be suitable for this task.[^30^, ^31^, ^32^, ^33^, ^34^, ^35^, ^36^, ^37^, ^38^, ^39^] We decided to select acetonitrile as elution solvent due to the fact that it is advantageous for the chromatography, especially for peak shapes, when the composition of the sample solvent of the final sample is identical with that of the mobile phase of the employed LC‐ESI‐MS/MS method which in the present case consists of a mixture of acetonitrile and 5 mM ammonium bicarbonate buffer in a ratio of 80 : 20 (*v*/*v*). For the final sample this solvent composition can be achieved by just adding a defined small volume of 5 mM ammonium bicarbonate buffer (pH 7.8). This leads only to a slight marker dilution from the original concentration to 80 % which can be easily taken into account in the quantification procedure.

During method development it was further recognized that Org25543 is prone to extensive adhesion to container materials when it is dissolved in pure water, especially to polypropylene surfaces of tubes, which leads to a decrease of the free concentration of the analyte as compared to the nominal concentration. This phenomenon has already been observed during method development in other studies.[^35^, ^43^] To solve this problem we used, as successfully done in former cases, *N,N*‐dimethylacetamide (DMA) as organic co‐solvent. A concentration of 10 % DMA (*v*/*v*) in water was already enough to gain satisfying results. Thus, all working solutions of Org25543 were prepared in 10 % aqueous DMA (Figure S4). As a consequence thereof, DMA was also contained in the final binding samples of the MS Binding Assays. Though this could have influenced ligand affinities, DMA was found to not effect Org25543 binding up to concentrations of 2 % ($\hat{=}$ 215 mM; Figure S5). As DMA concentration in binding samples was limited to 0.4 %, a negative effect could be clearly excluded.

Another important question was how to determine nonspecific binding of Org25543 in binding experiments. To this end, three different approaches were investigated which are commonly applied for this purpose. Firstly, nonspecific binding was evaluated over a concentration range from 0.4 to 150 nM (according to the concentrations in saturation experiments), by applying an excess of the cyclohexane analogue **8** of Org25543 as competitor (Figure 1, **8**), which is a known GlyT2 inhibitor (IC_50_=84 nM^[23]^), too. Secondly, it was studied with GlyT2 membrane fragments, which had been treated at 60 °C for 1 h in a water bath so that the proteins can be expected to be denatured (“heat‐shock”) and, thirdly, with membrane fragments of non‐transfected HEK293 cells. Comparing the results of the three afore described approaches it turned out that using **8** leads to a lower nonspecific binding than using heat‐shocked target material or empty HEK293 cells, which was found to be due to the fact that **8** also reduced filter binding of Org25543 to some extent (for details, see Figure S6). For the heat‐shocked target material and the empty HEK293 cells, nonspecific binding was identical. Clearly, the use of the same membrane preparation for determination of total and nonspecific binding as it is the case in the heat‐shock approach appears more appropriate than using different membrane fragments, that is, from transfected and non‐transfected HEK293 cells. As the former approach has already been used successfully in another project,^[44]^ we decided to apply this method for determination of nonspecific binding of Org25543 here, too.

Based on the above‐mentioned findings and on data already published for MS Binding Assays, the following conditions were established for the performance of the first binding assay addressing GlyT2 (see the scheme describing the workflow depicted in Figure 3). For binding Org25543 as reporter ligand is incubated with crude membrane fractions of HEK293 cells expressing GlyT2 for 1 h at 37 °C in 96‐deepwell plates in incubation buffer (10 mM HEPES, 120 mM NaCl, 2 mM KCl, 1 mM MgCl_2_, 1 mM CaCl_2_; pH 7.5) in a total volume of 250 μL. After the incubation aliquots of 210 μL of the corresponding binding samples are transferred to 96‐well filter plates (pretreated with PEI; for further information, see the Experimental Section) for the separation of target‐marker complexes from the liquid part of the incubation mixture and contained unbound marker by vacuum filtration. For the removal of remaining unbound reporter ligand, the filters are washed subsequently to the filtration with ice‐cold aqueous ammonium acetate buffer (4×150 μL; 154 mM; pH 7.4). After the filter plates with the remaining target‐marker complexes have been dried at 50 °C for 1 h, bound Org25543 is released from the target‐marker complex by eluting it with a [^2^H_7_]Org25543 solution in acetonitrile (3×70 μL; 125 pM) into a 96‐deepwell receiver plate by vacuum filtration. To adjust the sample solvent to the mobile phase, aqueous ammonium bicarbonate buffer (52.5 μL; 5 mM; pH 7.8) is added to each sample leading to the same solvent composition as in the mobile phase (buffer/acetonitrile 20 : 80, *v*/*v*). The thus obtained samples are finally subjected to an LC‐ESI‐MS/MS analysis according to the developed LC‐ESI‐MS/MS method (45 μL injection volume) for the quantification of the reporter ligand Org25543.

### 2.4 LC‐ESI‐MS/MS method validation

After the technical details for the performance of the MS Binding Assays had been established, including the generation of a defined matrix the developed LC‐ESI‐MS/MS method was ready to be validated for linearity, lower limit of quantification (LLOQ), accuracy, precision and selectivity according to the recommendation of the FDA guidance for bioanalytical method validation.^[45]^ For this purpose at first a blank matrix was prepared according to the above described procedure for the binding experiments and the corresponding blanks, standards and quality controls (QC) created therefrom were used to examine the developed LC‐ESI‐MS/MS method regarding the mentioned parameters. For preparing the blank matrix the incubation of target and buffer was performed without reporter ligand or any other test compound. To investigate the validation parameters, matrix calibration standards and quality control samples, matrix blanks and zero samples were prepared on different days with different GlyT2 membrane preparations in five sets, which contained Org25543 in a concentration range from 5 pM to 1 nM (except for matrix blanks and zero samples) as well as the internal standard [^2^H_7_]Org25543 in a fixed concentration of 100 pM (except for matrix blanks, for details see the Experimental Section). The established sets of calibration standards, quality control samples, blanks and zero samples were analyzed by using the developed LC‐ESI‐MS/MS method. The validation results showed that this method is in full agreement with the criteria from the FDA guidance regarding linearity in a range from 5 pM to 1 nM, intra‐ and inter‐batch precision and accuracy for QC samples at four different concentration levels (LLOQ, 15, 300, and 800 pM; defined criteria for linearity, intra‐ and inter‐batch precision and accuracy as given in the FDA guidance; see the Experimental Section). Overall, in five validation series we determined for the calibration standards accuracies between 94.2–108.6 % whereas the QC samples exhibited accuracies and precisions (expressed in relative standard deviations) between 96.3–110.0 % and 0.7–5.3 % for intra‐batch and 100.8–103.9 % and 3.4–5.2 % for inter‐batch, respectively. Selectivity was examined by injecting six individually prepared matrix blanks per validation series to the LC‐MS/MS. Thereby, no interfering signals were detected for the chosen mass transitions of the reporter ligand and the internal standard, thus the criteria of selectivity is fulfilled. The results of all sets obtained during the validation regarding linearity, precision and accuracy are summarized in Supporting Information (Table S1). Representative chromatograms for Org25543 at the LLOQ and a matrix blank and the linear calibration function received from one set of calibration standards are depicted in Figure 4. The here presented results are all in accordance with the defined criteria, thus it was demonstrated that the developed LC‐ESI‐MS/MS method is reliable for the quantification of Org25543 as reporter ligand in MS Binding Assays.

### 2.5 GlyT2 MS Binding Assays: Kinetic experiments

Next, the validated LC‐ESI‐MS/MS method should be employed for quantification of Org25543 in binding experiments. For the generation of reliable results in saturation and competitive experiments it has, however, to be warranted that the reporter ligand reaches equilibrium binding. Since no information about binding kinetics for any GlyT2 inhibitor are available so far, we decided to start our GlyT2 MS Binding Assays experiments with the determination of the off‐ and on‐rate (*k*_off_ and *k*_on_) of Org25543 at GlyT2 in dissociation and association experiments. That way also the equilibrium dissociation constant (*K*_d_) can be obtained, that is, by calculation from the kinetic rate constants. Furthermore, dissociation experiments were expected to shed some light on the binding characteristics of Org25543. In electrophysiological oocyte glycine current assays, it had namely been found that GlyT2 function cannot be restored completely, when cells are washed for several minutes to remove Org25543, which points towards a tight binding of Org25543 to GlyT2 resulting in a very slow off‐rate.[^11^, ^21^] Hence, we first performed dissociation experiments and after that association experiments.

The kinetic experiments were performed under conditions described above (see Section 2.3.) except that the incubation was done in bulk form. For further information about the setup of the kinetic experiments, see Experimental Section.

For the determination of the dissociation rate constant of Org25543 the so‐called displacer method was applied. Thus, after the target protein GlyT2 had been incubated with the reporter ligand Org25543 at a defined concentration (final concentration: 10 nM) and the binding allowed to reach equilibrium (1 h) dissociation was initiated by addition of **8** (final concentration: 30 μM). Overall, 12 samples were taken, the first after 15 s and the last after 3 h, and processed as described for competitive binding experiments. The same way nonspecific binding was determined except that the target protein had been subjected to a heat‐shock before. Plotting of specific binding calculated from total and nonspecific binding against time yielded the respective dissociation curves. Therefrom, by means of nonlinear regression analysis a *k*_off_ of 7.07±0.26×10^−3^ s^‐1^ (mean±SEM, *n*=3) and a half‐life (*t*_1/2_) of 98.4±3.5 s (mean±SEM, *n*=3) could be deduced. In Figure 5a, a representative dissociation curve of the specific binding of Org25543 is depicted. The half‐life of Org25543 at 37 °C showed that it is long enough to avoid unwanted dissociation effects during the washing procedure in the workflow of the GlyT2 MS Binding Assays. Furthermore, these results clearly demonstrate that Org25543 is a fully reversible ligand at GlyT2. No extremely tight binding or slow off‐rate towards GlyT2 can be observed. In this context, however, it is worth mentioning that the existence of covalent binding of Org25543 at GlyT2 to some extent, though very unlikely, cannot be absolutely excluded, as the amount of covalent binding that would have to be quantified by the established MS Binding Assays might be beyond its capability.

Next, for the determination of the association kinetics of Org25543 association experiments had to be performed. By the knowledge of [L] and *k*_off_, from *k*_obs_ as the primary result of such experiments, finally, the derived rate constant *k*_on_ becomes accessible by using the relation given at Equation 1, the transformed form of which is Equation 2.

(1) $$k{}_{\mathrm{obs}}=k{}_{\mathrm{on}}\times L+k{}_{\mathrm{off}}\;$$

(2) $$k{}_{\mathrm{on}}=\left(k{}_{\mathrm{obs}}-k{}_{\mathrm{off}}\right)/L$$

For the determination of *k*_obs_ Org25543 was added to the target protein, to start the association process, which was then stopped at eleven different time points (15 s–2 h), to measure the amount of bound marker at these defined time points. The nonspecific binding was determined for the same time points like for total binding samples but utilizing target material that had been subjected to a heat‐shock before. The concentration of the reporter ligand Org25543 was set to a value of 10 nM. This value was low enough to ascertain an association kinetic, that was slow enough to be reliably monitored by the applied technique. For the establishment of association curves specific binding calculated from total binding and nonspecific binding was plotted against time. By means of nonlinear regression analysis of these curves a *k*_obs_ value of 1.72±0.01×10^−2^ s^−1^ (mean±SEM, *n*=3) was obtained. Therefrom, with the *k*_off_ value determined before and the known concentration of Org25543 that had been applied, a *k*_on_ of 1.01×10^6^ M^−1^ s^−1^ has been calculated. A representative association curve of the specific binding of Org25543 to GlyT2 is depicted in Figure 5b.

The determined dissociation and association rate constants were finally used to calculate the equilibrium dissociation constant (*K*_d_) of Org25543 at GlyT2 according to the equation *K*_d_=*k*_off_/*k*_on_ which yielded a value of 6.99 nM.

As this is the first binding assay for GlyT2 no binding affinities from other binding assays are available for comparison purposes. So far Org25543 has been characterized only with IC_50_ values of 16±1.9 nM in a [^3^H]glycine uptake assay^[23]^ and 31±6 nM in a FLIPR membrane potential assay.^[27]^ Although these assays are based on distinctly different principles and the inhibitory potencies obtained thereby cannot be expected to be identical with the affinity constant from a binding assay, that is, the *K*_d_ value of Org25543 found in this GlyT2 MS Binding Assays, these data still clearly indicate that the *K*_d_ value found in this study is in a plausible order of magnitude.

### 2.6 GlyT2 MS Binding Assays: Saturation experiments

For the further characterization of the binding of Org25543 to GlyT2 saturation experiments were performed. To determine total binding of Org25543 GlyT2 membrane fractions were incubated with the reporter ligand in eight different concentrations (0.4–150 nM) and the samples analyzed after the general workup by LC‐ESI‐MS/MS as described above. Nonspecific binding was obtained from experiments that were completely analogous to the former except that protein subjected to a prior heat‐shock was used. Quantification of Org25543 in total binding samples could be realized down to the lowest nominal concentration level (0.4 nM). For nonspecific binding samples the quantification was possible down to 2 nM nominal marker concentration. For nominal marker concentrations lower than 2 nM the concentrations of Org25543 lay below the LLOQ. As it is generally acknowledged that nonspecific binding is related to nominal marker concentration in a linear fashion,^[46]^ data points below this concentration were calculated by extrapolation after linear regression analysis of the data points for nonspecific binding at nominal marker concentrations from 2 to 150 nM.

A representative curve obtained from a saturation experiment with Org25543 is depicted in Figure 6. It shows total and nonspecific binding as well as the specific binding of Org25543 towards GlyT2. By means of nonlinear regression analysis a saturation isotherm was generated from the data points representing specific binding, which revealed a *K*_d_ value of 7.45±0.55 nM (mean±SEM, *n*=3) and a maximum density of binding sites (*B*_max_) of 26.15±1.03 pmol (mg protein)^−1^ (mean±SEM, *n*=3). The thus obtained *K*_d_ value is clearly in excellent accord with the *K*_d_ value obtained for Org25543 from the kinetic experiments (7.45 vs. 6.99 nM).

### 2.7 GlyT2 MS Binding Assays: Competition experiments

As already mentioned above, there is a strong need for competitive binding assays addressing GlyT2 as a tool for the identification of new GlyT2 inhibitors as well as the characterization of the binding affinities of the respective ligands. For the development of the desired competitive MS Binding Assay, the general concept of this kind of binding experiments was followed, in which the affinity of a test compound is delineated from its potency to compete out a reporter ligand, in our case Org25543, from its target binding site. Accordingly, the target protein was incubated with a defined concentration of the reporter ligand and increasing concentration of the individual test compound. In detail, the competitors were studied at seven different concentration levels, which covered about three concentration log units, whereas the concentration of Org25543 was fixed to 10 nM. Thereafter, via the developed LC‐ESI‐MS/MS method the amount of bound marker (total binding) was determined for the samples obtained from the individual incubation mixtures by the common workup for MS Binding Assays. Nonspecific binding was again determined in a set of analogous experiments in which GlyT2 membrane fractions were replaced by material subjected to a heat‐shock before. The resulting amount of Org25543 binding was defined as 0 % level of specific reporter ligand binding. Plotting the percentage of bound marker (*y*‐axis) against the logarithm of the competitor concentration (*x*‐axis) yielded the corresponding sigmoidal competition curves, from which by means of nonlinear regression analysis the IC_50_ values of the studied test compounds could be obtained. The thus determined IC_50_ values were finally used to calculate the corresponding *K*_i_ values by means of the Cheng‐Prusoff equation.

In total 19 compounds were investigated including glycine, glycine derivatives and a series of small amino acids already characterized at GlyT1 and GlyT2 in other biological assays. Furthermore, a set of commercially available GlyT2 inhibitors, including representatives of a new class of lipid‐based GlyT2 inhibitors, as well as selective GlyT1 inhibitors and antipsychotics the characterization of which at GlyT2 has been reported in literature, have been studied for their ability to inhibit Org25543 binding. All these compounds are shown in Figure 1. The results of the competitive experiments are listed in Table 1, representative competition curves are depicted in Figure 7. As no data from other binding assays for GlyT2 is available so far, the obtained results were compared as far as possible with biological activities/potencies published for functional assays in literature.

For the glycine derivatives sarcosine [**9**] and glycine methyl ester [**10**] and for the amino acids dl‐proline [(*rac*)‐**11**], l‐serine [(*S*)‐**12**] and dl‐leucine [(*rac*)‐**13**] results were obtained which are largely in agreement with potencies determined in [^3^H]glycine uptake assays at GlyT2.[^22^, ^47^] For these compounds either no inhibition of Org25543 binding to more than 50 % could be found up to mM concentrations of the compounds (sarcosine, l‐serine, dl‐leucine) or the determined *K*_i_ value lies in the high‐millimolar range (glycine methyl ester p*K*_i_=1.11±0.06; dl‐proline p*K*_i_=1.50±0.11). However, for glycine [**1**], the actual substrate of GlyT2, the determined *K*_i_ value of about 10 mM (p*K*_i_=1.98±0.08) found in our GlyT2 MS Binding Assays is noticeable high. In literature a broad range of Michaelis‐Menten constants (*K*_m_) and EC_50_ values have been published (6–1801 μM),[^11^, ^18^, ^22^, ^27^, ^47^, ^48^, ^49^, ^50^, ^51^] reflecting that GlyT2 mediated transport of glycine may strongly depend on the chosen experimental conditions in the respective functional experiments. Though no satisfactory explanation for the rather low affinity determined for glycine at GlyT2 in the MS Binding Assay can be given, it should still be mentioned that a similar tendency (i. e., *K*_i_ in binding experiments > *K*_m_) has also been observed for GAT1 and GlyT1 in previous studies.[^29^, ^38^] However, in preliminary experiments, in which saturation of Org25543 at GlyT2 was studied in the presence of glycine, we obtained results that point to a competitive binding behavior between Org25543 and glycine (Figure S7 and Table S2). Possibly, Org25543 binds to a binding site different from the substrate binding site that is addressed by glycine as well, but with low affinity only. This could explain the difference that has been observed between potency and affinity of glycine at GlyT2. Nevertheless, MS Binding Assays are a valuable tool for the determination of binding affinities for test compounds towards GlyT2, the purpose they have been developed for, though they are not able to provide detailed information about the binding sites or binding mechanisms of test compounds. Another interesting aspect when looking at the inhibition curve of glycine is that it cannot completely displace Org25543 from its binding site even at the highest investigated concentration of 1 M. This can also be seen for dl‐proline (Figure S8). One possible explanation for this phenomenon could be that Org25543 binds to two different binding sites, of which only one can be addressed by glycine. However, neither in saturation nor in kinetic experiments any indications for a biphasic nature of the respective curves could be noted. Still, it has to be considered that to distinguish between different binding sites of the reporter ligand at GlyT2 in the generated binding curves were only possible, if the corresponding affinities or the kinetic behavior were markedly different. Another explanation for the observed phenomenon might be, that the high concentrations of glycine (or of structurally related compounds) of about 1 M cause unexpected effects by a so far unclear mechanism, which mediated an increase of nonspecific binding. In this context it has to be noted, that the bottom level of inhibition curves was in this case (and also in similar ones) due to the incomplete inhibition of Org25543 binding not constrained to the level of nonspecific binding as can easily be seen in Figure 7.

When the GlyT2 inhibitors ALX1393 [**3**], **8** and ZINC6865169 [**14**] were studied for ALX1393 a p*K*_i_ of 5.93 was found. This p*K*_i_ differs at least one order of magnitude from the pIC_50_ values reported in glycine uptake studies.[^11^, ^40^] Compound **8**, however, the analogue of Org25543 [**2**], exhibits a p*K*_i_ of 7.38 which lies in the same range as the published biological activity. Whereas **8** and Org25543 can be expected to have the same binding site due to their structural analogy, the binding site of ALX1393 could differ slightly from that of Org25543. Nevertheless, both inhibitors exhibited reasonable affinities in comparison to their potencies found in literature. More surprising are the findings for ZINC6865169, a recently described GlyT2 inhibitor with a structure significantly different from that of Org25543 and ALX1393. In glycine uptake studies an IC_50_ value of 518 nM was determined^[40]^ whereas the IC_50_ value in our GlyT2 MS Binding Assays was >600 μM (77 % remaining binding of Org25543 at 600 μM of ZINC6865169). Thus, for this compound there is a substantial difference between potency and affinity of more than three orders of magnitude, which could also argue for different bindings sites.

For the GlyT1 selective inhibitors Org24598 [(*R*)‐**15**], ALX5407 [(*R*)‐**16**], CP‐802,079 [(*rac*)‐**17**], ASP2535 [**18**] and LY2365109 [**19**] the results are in good agreement with published potencies. CP‐802,079 and ASP2535 exhibit p*K*_i_ values of 4.64 and 5.99, respectively, whereas Org24598 and LY2365109 show hardly any affinity towards the Org25543 binding site on GlyT2 up to concentrations of 300 and 10 μM, respectively. For ALX5407 two different inhibitory potencies have been reported in literature, i. e. an IC_50_ value>100 μM^[54]^ and an IC_50_ value of 1.8 μM.^[40]^ In the GlyT2 MS Binding Assay ALX5407 showed hardly a reduction of Org25543 binding up to 600 μM indicating its very low affinity. Though, it is not clear which of the two IC_50_ values of ALX5407 reported in literature is more reliable, the low affinity found for this compound in our MS Binding Assay (IC_50_>600 μM) fits at least quite well to the higher IC_50_ value, arguing for the validity of the result obtained in the MS Binding Assay.

The antipsychotics haloperidol [**20**] and chlorpromazine [**21**] are exhibiting moderate p*K*_i_ values of 4.90 and 4.55, respectively. These results are in very good agreement with the results published in literature (pIC_50_ of 4.89 and 4.68).

Finally, the most well‐known lipid‐based GlyT2 inhibitors *N*‐arachidonylglycine [**4**], *N*‐oleoylglycine [**5**] and oleoyl‐l‐carnitine [**22**] were tested in competition experiments. As already mentioned before, in the last years GlyT2 research was heading for inhibitors with better and safer pharmacological profiles. Thereby lipid‐based compounds were found, which are able to inhibit GlyT2 in a fully reversible manner and are partial inhibitors of this transporter, the latter of which is thought to positively contribute to the pharmacological profile of these compounds.[^17^, ^18^, ^19^, ^20^, ^21^] The affinities that were found in MS Binding Assays for the herein examined lipid‐based compounds are in the same range for all three substances (p*K*_i_ of 5.40, 5.63 and 5.58). Compared to their inhibitory potencies only *N*‐arachidonylglycine exhibits a p*K*_i_ value that is similar to the published pIC_50_ value (calculated from IC_50_), whereas the p*K*_i_ values of *N*‐oleoylglycine and oleoyl‐l‐carnitine are slightly lower than the pIC_50_ values calculated from the published potencies (less than one order of magnitude). So far, it is still a matter of discussion how these lipid‐based inhibitors are interacting with GlyT2. It is assumed that they either interact directly with GlyT2 at extracellular loops or at the interface between GlyT2 and the surrounding lipid membrane[^18^, ^59^] or that they are inhibiting GlyT2 indirectly by perturbing the biophysical properties of the bilayer surrounding GlyT2.^[60]^ Due to the difference of their structure in comparison to Org25543 it does not appear unlikely that these compounds bind to a binding site different from that of Org25543, which would explain why the determined binding affinities are different from the published potencies. Additionally, another point worth mentioning in this context, are the critical micelle concentrations (CMC) of the lipid‐based compounds. For *N*‐arachidonylglycine [**4**] and oleoyl‐l‐carnitine [**22**] CMCs of >100^[61]^ and 7.4 μM^[18]^ were published, respectively, whereas for *N*‐oleoylglycine [**5**] to the best of our knowledge no CMC has been described so far. Taking the knowledge about the CMCs into account the result obtained for oleoyl‐l‐carnitine [**22**] should be handled with care as the concentrations in competitive experiments are higher than the respective CMC. Possibly, the obtained binding data do not only reflect pure competitive interactions but also effects that result from micelle formation. Micelle formation might lead to a decrease of the free concentration of these lipid‐based compounds or formed micelles might have a direct effect on the GlyT2 protein to some extent. Since the CMC for *N*‐oleoylglycine [**5**] is not known, the same could be true for it, as well. A decreased free concentration of *N*‐oleoylglycine [**5**] due to micelle formation, for example, could be an explanation for the incomplete displacement of the reporter ligand Org25543 [**2**] in the competition experiment performed with **5** (Figure 7). For *N*‐arachidonylglycine [**4**], the highest employed concentrations were still below the mentioned CMC. Thus, in this case the formation of micelles in the binding assay is to be excluded, which might explain why binding data from the MS Binding Assay and data from functional assays (from literature) are in good accord.

Summarizing the findings resulting from the discussion above, it can be concluded that the affinities determined in the competition experiments are in fair to good agreement with inhibitory potencies obtained in various functional assays for the majority of the investigated compounds, but that there are also distinct discrepancies between affinity and inhibitory potency as exemplified for the GlyT2 inhibitor ZINC6865169. This outcome is in the end not too surprising, as the affinities measured in the established GlyT2 MS Binding Assays only record direct (i. e., competitive) and maybe additionally indirect (i. e., allosteric) interactions at the binding site labeled by Org25543, whereas inhibition in the various functional assays can furthermore be due to inhibition of binding sites not addressed by Org25543 or even by other mechanisms such as changes in protein expression or internalization – to name just a few.

## 3 Conclusion

The presented study describes the establishment of the first binding assays for the neurotransmitter transporter GlyT2. They are following the concept of MS Binding Assays and use the selective GlyT2 inhibitor Org25543 as unlabeled reporter ligand. For quantification of Org25543 in binding experiments a rapid and highly sensitive LC‐ESI‐MS/MS method was established. Validation of this method indicated that Org25543 can be accurately and precisely quantified in the matrix resulting from binding experiments in a range from 5 pM to 1 nM.

Based on the workflow of filtration‐based MS Binding Assays already established for other neurotransmitter transporters, binding experiments could be performed to characterize the binding behavior of Org25543 towards GlyT2 in kinetic, saturation and competitive experiments. In dissociation and association experiments the off‐ and on‐rate of Org25543 could be characterized with a *k*_off_ of 7.07×10^−3^ s^−1^ and a *t*_1/2_ of 98.4 s and with a *k*_obs_ of 1.72×10^−2^ s^−1^ resulting in a *k*_on_ of 1.01×10^6^ M^−1^ s^−1^, respectively. Furthermore, these experiments revealed full reversibility of Org25543 binding towards GlyT2 with an off‐rate that is not exceedingly slow. This result is noticeable as based on electrophysiological experiments with GlyT2 expressing oocytes Org25543 [**2**] was claimed to exhibit tight binding toward GlyT2 and to act like an irreversible inhibitor.[^11^, ^21^] Calculating the equilibrium dissociation constant from *k*_off_ and *k*_on_ yielded a value of 6.99 nM which matches perfectly the *K*_d_ of 7.45 nM determined in saturation experiments and which is roughly in agreement with potencies observed in functional assays. In competition experiments 19 known GlyT ligands were characterized for their affinity at the Org25543 binding site of GlyT2. For most of the investigated compounds affinities were determined that are largely in accordance with inhibitory potencies found in various functional assays. For few of the investigated compounds, inhibition of Org25543 binding did not parallel the inhibition described in functional assays. Besides differences in experimental conditions of the different assay techniques, possible explanations must remain speculative without extensive investigations. So far, it cannot be excluded, that occupation of different binding sites at GlyT2 contributes to this phenomenon, in particular when considering the novel extracellular allosteric modulator site for lipid‐based GlyT2 inhibitors just recently identified by Mostyn et al.^[62]^ in some cases, at very high concentrations of test compounds (e. g., for glycine) also solubility issues or formation of micelles (e. g., for lipid‐based GlyT2 inhibitors) might play a role. Apart from that it has to be pointed out, that the results from these binding experiments cannot be expected to be strictly and completely in agreement with inhibitory effects observed in various functional assays recording substrate transport, translocation of charges or membrane depolarization, as inhibition of functional effects recorded in all these assay types does not necessarily require occupation of the GlyT2 binding site addressed by Org25543.

In the context of this discussion, the established GlyT2 MS Binding Assays as well as all the different functional assay principles should be considered as valuable alternatives but not as absolutely equivalent substitutes, all of them possessing specific strengths and weaknesses. The strengths of binding assays are for example their conceptual simplicity, their robust read out and the possibility to derive structure‐activity relationships based on determined affinities in a very straightforward way. Therefore, the presented GlyT2 MS Binding Assays can be assumed to fill a gap and will hopefully contribute to facilitate screening for new GlyT2 inhibitors and furthermore, to assign GlyT2 inhibitors to different categories according to their binding behavior.

## Experimental Section

### Chemicals

4‐Benzyloxy‐3,5‐dimethoxy‐*N*‐[1‐[(dimethylaminocyclohexyl)methyl]benzamide was synthesized in‐house according to Caulfield et al.^[23]^ Org25543 (*N*‐[[1‐(dimethylamino)cyclopentyl]methyl]‐3,5‐dimethoxy‐4‐(phenylmethoxy)benzamide) as hydrochloride (purity ≥99 %, HPLC), Org24598 (*N*‐methyl‐*N*‐[(3*R*)‐3‐phenyl‐3‐[4‐(trifluoromethyl)phenoxy]propyl]glycine) as lithium salt (purity ≥98 %, HPLC), ALX5407 (*N*‐[(3*R*)‐3‐([1,1′‐biphenyl]‐4‐yloxy)‐3‐(4‐fluorophenyl)propyl]‐*N*‐methylglycine) as hydrochloride (purity ≥98 %, HPLC), and *N*‐arachidonylglycine (NAGly, *N*‐(1‐oxo‐(5*Z*,8*Z*,11*Z*,14*Z*)‐eicosatetraenyl)glycine; purity ≥98 %, HPLC) were purchased from Tocris (Bristol, UK). LY2365109 (*N*‐[2‐[4‐(1,3‐benzodioxol‐5‐yl)‐2‐(1,1‐dimethylethyl)‐phenoxy]ethyl]‐*N*‐methylglycine) and ASP2535 (4‐[3‐(1‐methylethyl)‐5‐(6‐phenyl‐3‐pyridinyl)‐4*H*‐1,2,4‐triazol‐4‐yl]‐2,1,3‐benzoxadiazole) were part of the Tocriscreen Plus library from Tocris (Bristol, UK). CP‐802,079 (*N‐*[3‐(4‐chlorophenyl)‐3‐[4‐(2‐thiazolylcarbonyl)phenoxy]propyl]‐*N*‐methyl‐glycine) as hydrochloride (purity ≥98 %), ALX1393 (*O*‐[(2‐benzyloxyphenyl‐3‐flurophenyl)methyl]‐l‐serine) (purity ≥98 %), haloperidol, chlorpromazine as hydrochloride (purity ≥98 %, TLC) and ZINC6865169 (5‐((8‐hydroxy‐9*H*‐purin‐6‐yl)thio)pentanoic acid) were purchased from Sigma‐Aldrich. Glycine (purity ≥99 %), dl‐proline (purity ≥99 %) and dl‐leucine (purity ≥99 %) were obtained from Acros Organics, glycine methyl ester as hydrochloride (purity ≥99 %) and l‐serine (purity ≥99 %) were from Merck and sarcosine as hydrochloride was from ICN Biomedicals (Irvine, CA, USA). Oleoyl‐l‐carnitine (OLCarn; purity >99 %, TLC) and *N‐*oleoylglycine (NOGly, *N*‐(1‐oxo‐9‐octadecenyl)‐(*Z*)‐glycine; purity ≥98 %) were received from Avanti Polar Lipids and Cayman Chemical Company (Ann Arbor, MI, USA). Water was exclusively obtained from a Sartorius arium pro ultrapure water system (Sartorius, Göttingen, Germany). HPLC and LC‐MS grade methanol from VWR Prolabo (Darmstadt, Germany) was used for washing the glass fiber filters and for determination of compound‐dependent MS parameters, respectively. LC‐MS grade acetonitrile from VWR Prolabo (Darmstadt, Germany) was used for elution of marker from target‐marker‐complexes and for the mobile phase in LC‐MS. All other chemicals were purchased in analytical grade. For cell culture, Dulbecco's modified Eagle's medium (DMEM) was bought from Sigma‐Aldrich), fetal bovine serum, penicillin and streptomycin were from BioWest (Nuaillé, France) and hygromycin B (Hygromycin B Gold) was obtained from InvivoGen.

### LC‐ESI‐MS/MS instrumentation

An API5000 triple quadrupole mass spectrometer with a TurboV‐ESI source (AB Sciex, Darmstadt, Germany) was used for the LC‐ESI‐MS/MS. As HPLC system an Agilent 1200 Series HPLC system (vacuum degasser G1379B, binary pump G1312B, oven G1316B, Agilent, Waldbronn, Germany) and a HTS‐PAL auto sampler (CTC‐Analytics, Zwingen, Switzerland) with a 50 μL syringe and a 50 μL sample loop was coupled to the mass spectrometer. For controlling the hardware components, the Analyst v. 1.6.1 software (AB Sciex, Darmstadt, Germany) was integrated.

### Compound‐dependent MS parameters for precursor and fragment ions of ALX1393, Org25543 and [^2^H_7_]Org25543

An external syringe pump (Harvard Apparatus, Holliston, MA, USA) set to a flow rate of 10 μL min^−1^ was used to infuse 20 nM solutions of ALX1393, Org25543 and [^2^H_7_]Org25543 in a mixture of methanol (LC‐MS grade) and 0.1 % (*v*/*v*) formic acid (LC‐MS grade; 50 : 50, *v*/*v*) into the ESI source. With the manual tuning mode of the Analyst software *m*/*z* 396,1, *m*/*z* 413.2 and *m*/*z* 420.3 were identified as [*M*+H]^+^ parent ions for ALX1393, Org25543 and [^2^H_7_]Org25543, respectively. To identify the most intense fragment ions (*m*/*z* 291.3 for ALX1393, *m*/*z* 368.3 for Org25543 and *m*/*z* 375.4 for [^2^H_7_]Org25543) as well as to optimize the compound‐dependent parameters for the precursor ions the compound optimization mode of the Analyst software was used. The optimized parameters are listed below: ALX1393: Declustering potential (DP) 66 V, entrance potential (EP) 10 V, collision energy (CE) 15 V, cell exit potential (CXP) 28 V; Org25543: DP 106 V, EP 10 V, CE 25 V, CXP 14 V; [^2^H_7_]Org25543: DP 91 V, EP 10 V, CE 27 V, CXP 14 V.

### Chromatography

An isocratic RP‐LC method was developed for chromatography, which used a mobile phase consisting of ammonium bicarbonate buffer (5 mM, pH 7.8) and acetonitrile (20 : 80, *v*/*v*) at a flow rate of 600 μL min^−1^ at 20 °C. As stationary phase a Luna 3 μ C8(2) column (50 mm×2 mm, 3 μm, Phenomenex, Aschaffenburg, Germany) was integrated into the system. A SecurityGuard C8 column (4 mm×2 mm, Phenomenex, Aschaffenburg, Germany) and two in‐line filters (0.5 and 0.2 μm, Idex, Oak Harbor, WA, USA) are installed before the column for protection reasons. The injection volume was set to 45 μL.

### LC‐ESI‐MS/MS

To analyze ALX1393, Org25543 and [^2^H_7_]Org25543 the mass transitions of *m*/*z* 396.1/291.3, *m*/*z* 413.2/368.3 and *m*/*z* 420.3/375.4 were used, respectively. Both mass selectors, Q1 and Q3, were operated under unit resolution for dwell times of 500 ms under the conditions mentioned in Experimental Section *Compound‐dependent MS parameters for precursor and fragment ions of ALX1393, Org25543 and [^2^H_7_]Org25543*. After Org25543 was selected as reporter ligand the optimized source‐dependent parameters were determined by using the flow injection analysis (FIA) tool of the Analyst software. For that a solution containing 100 pM of Org25543 and [^2^H_7_]Org25543 was injected (10 μL) to the LC‐MS/MS which resulted in following optimized parameters: source temperature 600 °C, ion‐spray voltage 2500 V, curtain gas (N_2_) 15 psi, auxiliary gas (N_2_) 40 psi, nebulizing gas (N_2_) 60 psi and collision gas (N_2_) 7 psi.

### Synthesis of methyl 3,5‐dimethoxy‐4‐(((2,3,4,5,6‐[^2^H_5_])phenyl)[^2^H_2_]methoxy)benzoate (25)

[^2^H_7_]benzyl chloride (**24**; 862 μL, 7.48 mmol) was added dropwise to the suspension of methyl syringate (**23**; 1.47 g, 6.80 mmol), K_2_CO_3_ (1.14 g, 8.16 mmol) and KI (1.13 g, 6.80 mmol) in DMF (10 mL) at RT. The reaction mixture was stirred at 80 °C for 24 h. After 24 h the reaction mixture was cooled to RT, then added to H_2_O (50 mL) and extracted with ethyl acetate (4×50 mL). The combined organic layers were washed with H_2_O (20 mL) and brine (20 mL) then dried (Na_2_SO_4_), filtered and concentrated *in vacuo* to afford 1.95 g (93 %) methyl 3,5‐dimethoxy‐4‐(((2,3,4,5,6‐[^2^H_5_])phenyl)[^2^H_2_]methoxy)benzoate (**25**) as colorless solid. ^1^H NMR (400 MHz, CD_2_Cl_2_): *δ*=7.29 (s, 2H, 2×HC_arom_), 3.88 (s, 3H, CH_3_OC=O), 3.87 (s, 6H, 2×CH_3_OC_arom_). ^13^C NMR (100 MHz, CD_2_Cl_2_): *δ*=167.06 (C=O), 153.86 (2×CH_3_O*C*_arom_), 141.49 (CH_3_OC*C*O), 137.98 (*C*C[^2^H_2_]), 128.8–127.6 (m, *5* C, 5×C[^2^H]_arom_), 125.94 (*C*C=O), 107.19 (2×HC_arom_), 74.51 (quint, *J*_C‐D_=22.3 Hz, C[^2^H_2_]), 56.69 (2×CH_3_OC_arom_), 52.57 (*C*H_3_OC=O). IR (KBr): $\tilde{\nu }$ =3400, 3101, 2980, 2949, 2874, 2844, 2638, 2270, 2207, 2120, 1950, 1931, 1711, 1591, 1499, 1467, 1437, 1414, 1332, 1227, 1185, 1129 cm^−1^. MS HRMS (EI): [*M*]^+.^ calcd. for C_17_H_11_[^2^H_7_]O_5_ 309.1594; found: 309.1588.

### Synthesis of 3,5‐dimethoxy‐4‐(((2,3,4,5,6‐[^2^H_5_])phenyl)[^2^H_2_]methoxy)benzoic acid (26)

To a stirred solution of **25** (890 mg, 2.88 mmol) in a mixture of THF (6 mL) and H_2_O (2 ml) was added LiOH (352 mg, 14.4 mmol) at RT. After 68 h the reaction mixture was poured onto H_2_O (20 mL) followed by the addition of HCl (1 M, 15 mL). The acidic aqueous solution (pH 1–2) was extracted with ethyl acetate (5×30 mL). The combined organic layers were dried (Na_2_SO_4_), filtered and concentrated *in vacuo* to afford 808 mg (95 %) 3,5‐dimethoxy‐4‐(((2,3,4,5,6‐[^2^H_5_])phenyl)[^2^H_2_]methoxy)benzoic acid (**26**) as a colorless solid. ^1^H NMR (400 MHz, CD_2_Cl_2_): *δ*=7.37 (s, 2H, 2×HC_arom_), 3.89 (s, 6H, 2×CH_3_OC_arom_). ^13^C NMR (100 MHz, CD_2_Cl_2_): *δ*=172.31 (C=O), 153.95 (2×CH_3_O*C*_arom_), 142.45 (CH_3_OC*C*O), 137.88 (*C*C[^2^H_2_]), 128.49 (t, *J*_C‐D_=24.2 Hz, 2×[^2^H]C_arom_), 128.22 (t, *J*_C‐D_=24.2 Hz, 2×[^2^H]C_arom_), 128.03 (t, *J*_C‐D_=24.2 Hz, [^2^H]C_arom_), 124.68 (*C*C=O), 107.87 (2×HC_arom_), 74.58 (quint, *J*_C‐D_=22.1 Hz, C[^2^H_2_]), 56.74 (2×CH_3_OC_arom_). IR (KBr): $\tilde{\nu }$ =3445, 2997, 2962, 2836, 2271, 2208, 2162, 2120, 1682, 1589, 1500, 1450, 1416, 1327, 1278, 1231, 1200, 1184, 1128 cm^−1^. MS HRMS (EI): [*M*]^+.^ calcd. for C_16_H_9_[^2^H_7_]O_5_ 295.1437; found: 295.1436.

### Synthesis of [^2^H_7_]Org25543 ⋅ HCl (7 ⋅ HCl)

To a stirred solution of 1‐(aminomethyl)‐*N*,*N*‐dimethylcyclopentane‐1‐amine^[41]^ (353 mg, 1.49 mmol) in CH_2_Cl_2_ (20 mL) was added **26** (400 mg, 1.35 mmol) at RT followed by the addition of benzotriazole‐1‐ol (HOBt; 187 mg, 1.35 mmol), 3‐(ethyliminomethyleneamino)‐*N*,*N*‐dimethylpropan‐1‐amine (EDCI; 265 mg, 1.35 mmol) and NEt_3_ (137 mg, 1.35 mmol). The resulting reaction mixture stirred at RT for 18 h then poured on H_2_O (50 mL) followed by the extraction with ethyl acetate (2×50 mL). The combined organic layers were washed with HCl (1 M, 10 mL), H_2_O (10 mL) and brine (10 mL), then dried (MgSO_4_), filtered and concentrated *in vacuo* to afford 400 mg (70 %) [^2^H_7_]Org25543 (**7**) as a yellowish oil. Finally, to a stirred solution of **7** in diethyl ether HCl (4 M, 250 μL) in dioxane was added at 0 °C under gently shaking. The resulting colorless precipitate was filtered, washed with diethyl ether and dried *in vacuo* to obtain 395 mg (91 %) **7**⋅HCl. ^1^H NMR (500 MHz, [D_4_]methanol): *δ*=7.26 (s, 2H, 2×HC_arom_), 3.89 (s, 6H, 2×CH_3_O), 3.76 (s, 2H, NCH_2_), 2.97 (s, 6H, 2×CH_3_N), 2.11–1.80 (m, 8H, 8×cyclopentyl‐H). ^13^C NMR (126 MHz, [D_4_]methanol): *δ*=170.90 (C=O), 154.80 (2×CH_3_O*C*_arom_), 141.10 (CH_3_OC*C*O), 138.45 (*C*C[^2^H_2_]), 130.02 (*C*C=O), 129.24 (t, *J*_C‐D_=24.0 Hz, 2×[^2^H]C_arom_), 128.64 (t, *J*_C‐D_=24.4 Hz, 2×[^2^H]C_arom_), 128.60 (t, *J*_C‐D_=24.2 Hz, [^2^H]C_arom_), 106.30 (2×HC_arom_), 77.35 (N*C*CH_2_), 75.11 (quint, *J*_C‐D_=22.1 Hz, C[^2^H_2_]), 56.90 (2×CH_3_O), 43.31 (*C*H_2_NC=O), 40.07 (2×CH_3_N), 33.53 (2×*C*H_2_CN), 25.37 (2×*C*H_2_CH_2_CN). IR (film): $\tilde{\nu }$ =3434, 3234, 2994, 2964, 2876, 2842, 2668, 2595, 2516, 2478, 2279, 2201, 2117, 1669, 1587, 1531, 1496, 1414, 1332, 1272, 1225, 1194, 1167, 1124 cm^−1^. MS HRMS (ESI): [*M*+H]^+^ calcd. for C_24_H_25_[^2^H_7_]N_2_O_4_ 420.2880; found: 420.2876.

### Validation of the LC‐ESI‐MS/MS method

The validation of the developed LC‐ESI‐MS/MS method was oriented towards the FDA guidance for bioanalytical method validation.^[45]^ For this purpose, spiked matrix samples were used to examine the validation parameters linearity, LLOQ, precision, accuracy and selectivity. As described in Experimental Section *MS Binding Assay: General procedure* spiked matrix samples are obtained in the same way as binding samples are prepared, except for incubation which was carried out without marker or test compound and the elution step was performed with acetonitrile containing 125 pM [^2^H_7_]Org25543 and Org25543 in different concentrations. Those elution solutions were prepared from 125‐fold Org25543 and [^2^H_7_]Org25543 solutions in 10 % aqueous *N,N*‐dimethylacetamide (DMA) which were two times diluted 1 : 10 in acetonitrile (final DMA concentration 0.2 %). Five series of samples (matrix calibration standards and quality control (QC) samples, matrix blanks and zero samples) were prepared on different days with different GlyT2 membrane preparations. Calibration standards were studied at eight different concentration levels (5 pM to 1 nM). Every concentration level above the lower limit of quantification (LLOQ) was prepared in three replicates, whereas the concentration level of the LLOQ (5 pM) was prepared in six replicates. According to the FDA guidance the response of the LLOQ should be at least five times the response of the noise of a matrix blank, with an accuracy between 80 and 120 % and a precision characterized by a relative standard deviation of less than 20 %, whereas the accuracy of all other calibration standards and QC samples should be between 85 and 115 % and the precision of QC samples exhibit a relative standard deviation of not more than 15 %. The data for the calibration standards were obtained by plotting the peak area ratios of analyte vs. internal standard (*y*‐axis) against the concentration ratios of analyte vs. internal standard (*x*‐axis). Finally, linearity was studied after linear regression analysis of these data. For calculation of calibration curves a weighting factor of 1/*x* ^2^ was used in all cases. For examination of accuracy and intra‐ and inter‐batch precision QC samples were prepared independently from each other in different wells of a 96‐well plate (1.2 mL well volume, Sarstedt, Nümbrecht, Germany). Each series investigated four concentration levels (LLOQ, 15, 300, 800 pM) in six replicate QC samples. Selectivity of marker and internal standard was proven by injecting six matrix blanks in every series.

### Glycine transporter 2 (GlyT2) membrane preparations

For preparation of GlyT2 membrane preparations HEK293 cells, stably expressing the hGlyT2 with a confluence ≥90 %, were used (kindly provided by AbbVie (Wiesbaden, Germany)). As cell culture medium DMEM containing 10 % fetal bovine serum (*m*/*v*), 100 U mL^−1^ penicillin, 100 μg mL^−1^ streptomycin and 100 mg mL^−1^ hygromycin B was used and the cells were cultivated in dishes (143 cm^2^) at 37 °C and 8 % CO_2_. The cells were detached from the dish by aspirating the medium, adding 12 mL cell culture medium without hygromycin B and pipetting the medium several times until the cell lawn was completely detached. Subsequently, the cells were washed twice with PBS (5 min, 1600 rpm, Biofuge Stratos, rotor: #3047, Heraeus, Hanau, Germany) and afterwards homogenized in incubation buffer (10 mM HEPES, 120 mM NaCl, 2 mM KCl, 1 mM MgCl_2_, 1 mM CaCl_2_, pH 7.5) using a Polytron PTA 10 S (Kinematica Polytron, Littau‐Luzern, Switzerland). After homogenization aliquots of about 1 mg protein were frozen and stored at −80 °C. To determine the amount of protein in the membrane preparation (after treatment with 100 mM NaOH for 1 h) the protein determination according to Bradford^[63]^ was applied, using bovine serum albumin as standard for calibration. At the day of the assay the membrane preparation was rapidly thawed, diluted in 20 mL incubation buffer and centrifuged at 4 °C for 20 min at 20 500 rpm (Sorvall Evolution RC, rotor: SS34, Thermo. Electron, Hanau, Germany). Finally, the resulting pellet was resuspended in 6 mL incubation buffer yielding a protein concentration of ∼0.1–0.2 mg mL^−1^.

### MS Binding Assay: General procedure

In polypropylene 96‐well plates (1.2 mL well volume, Sarstedt) membrane preparations (∼5–10 μg protein) were incubated in the presence of the reporter ligand and, if required, test compounds in incubation buffer (10 mM HEPES, 120 mM NaCl, 2 mM KCl, 1 mM MgCl_2_, 1 mM CaCl_2_, pH 7.5) in a total volume of 250 μL at 37 °C in a Julabo SW‐20 C water bath (Julabo GmbH, Seelbach, Germany) for 1 h (every sample was prepared in triplicates). After the incubation 210 μL aliquots of the binding samples were transferred by means of a 12‐channel pipette onto 96‐well glass fiber filter plates (AcroPrep Advance, glass fiber, 1.0 μm, 350 μL; Pall Corporation, Port Washington, NY, USA) where the incubation was terminated by vacuum filtration (the samples were transferred and the incubation was terminated row after row; Multi Well Plate Vacuum Manifold, Pall, Dreieich, Germany). Before the binding samples were filtered, the glass fiber filters were washed with water (3×200 μL per well) and methanol (3×200 μL per well), incubated with 0.5 % (*m*/*m*) aqueous polyethylenimine solution (PEI; 200 μL per well) at room temperature for 2 h and, finally, exempted from the PEI solution by vacuum filtration. After the filtration of the binding samples, the filters with remaining target‐marker complexes on it were rapidly washed concurrently (12‐channel pipette) with ice‐cold washing buffer (4×150 μL 154 mM ammonium acetate, pH 7.4) and the filter plates were dried at 50 °C for 1 h. Subsequently, the bound marker was eluted with acetonitrile containing 125 pM [^2^H_7_]Org25543 and 0.2 % DMA (3×70 μL per well; in accordance to the calibration standards and QC samples; see Section 4.9.) into a 96‐well filter plate (1.2 mL well volume, Sarstedt; filtration via vacuum application after exposure of the filters of all wells to acetonitrile for ∼10 s; 12‐channel pipette). After the elution 52.5 μL ammonium bicarbonate buffer (5 mM, pH 7.8) were added per well (12‐channel pipette) to the eluate to adjust the composition of the sample solvent to the mobile phase (ammonium bicarbonate buffer (5 mM, pH 7.8) and acetonitrile 20 : 80, *v*/*v*). This means that the injected samples were diluted to 80 % of their actual concentrations. The 96‐well plates containing the eluted marker were sealed with aluminum foil and centrifuged 10 min at 2500 rpm (Biofuge Stratos, rotor: #3048, Heraeus). Finally, the samples were subjected to LC‐ESI‐MS/MS quantification (see above) without further sample preparation. In the same way nonspecific binding was determined by adding GlyT2 membrane fractions to the incubation samples, which were heat‐shocked before for 1 h at 60 °C in a water bath. Marker depletion was negligible (<10 %) in all experiments. DMA concentrations in final binding samples were always≤1 %.

### Saturation experiments

For saturation experiments GlyT2 membrane preparations were incubated with eight different concentrations of Org25543 in a concentration range of 0.4 to 150 nM. The following steps after incubation were performed as described in Experimental Section *MS Binding Assay: General procedure*. Nonspecific binding was determined in the same way as total binding but GlyT2 membrane fractions were used, which were heat‐shocked for 1 h at 60 °C, for the same marker concentrations. When nonspecific binding of Org25543 fell below the LLOQ of the calibration curve (generally at a nominal marker concentration of <2 nM), a straight line for the data points of nonspecific binding higher than the LLOQ was generated by using linear regression analysis and the nonspecific binding for data points below the LLOQ was calculated by extrapolation of the linear function using Prism v. 6.07 (GraphPad Software).

### Competition experiments

For competition experiments GlyT2 membrane preparations were incubated with Org25543 in a concentration of 10 nM and in presence of test compounds (at least seven concentrations). Additionally, control samples were prepared to define total binding of Org25543 in absence of any competitor and nonspecific binding was defined as remaining binding in presence of GlyT2 membrane preparations, which were heat‐shocked for 1 h at 60 °C. The top and bottom level of competition curves, which were showing a complete displacement of Org25543, were constrained to 100 and 0 %, respectively, whereas in competition curves, which were exhibiting incomplete inhibitions, only the top level was set to 100 % and the bottom‐level was not constrained.

### Kinetic studies: Dissociation experiments

Dissociation experiments via displacing Org25543 from GlyT2 with an excess of competitor were performed in 25 mL round bottom flasks made of glass (Duran Wheaton Kimble Life Sciences GmbH, Wertheim, Germany). For determining dissociation rate constants by the displacer approach GlyT2 membrane fractions were pre‐incubated with Org25543 at a nominal concentration of 10 nM at 37 °C in a water bath for 1 h in a total volume of 24.85 mL. The solution and the water bath were stirred constantly with magnetic stir bars at a frequency of ∼500 rpm. After 1 h of incubation dissociation was initiated by adding 150 μL of **8** resulting in 12 concentrations of 30 μM. After defined time points (15 s–3 h, twelve dissociation time points, samples for each time point were prepared in triplicates) dissociation was terminated via vacuum filtration and the samples were treated the same way as described in Experimental Section *MS Binding Assay: General procedure*. Nonspecific binding was determined at the same time points as for the dissociation samples but incubating 10 nM Org25543 in the presence of 30 μM **8** and heat‐shocked GlyT2 membrane preparations (triplicate).

### Kinetic studies: Association experiments

Association experiments were performed in 25 mL round bottom flasks made of glass (Duran Wheaton Kimble Life Sciences GmbH, Wertheim, Germany). For determining association rate constants Org25543 were added to the incubation buffer containing GlyT2 membrane preparations which results in a final concentration of 10 nM Org25543 and in a total volume of 25 mL. The binding solution was incubated at 37 °C in a water bath and both, binding solution and water bath, are stirred with magnetic stir bars at a frequency of ∼500 rpm. The incubation was terminated via vacuum filtration after defined time points (15 s–2 h, eleven association time points, samples for each time point were prepared in triplicates). The filtrated samples were treated the same way as described in Experimental Section *MS Binding Assay: General procedure*. Nonspecific binding at a marker concentration of 10 nM Org25543 was determined for the same time points as for the association samples but in the presence of heat‐shocked GlyT2 membrane preparations (triplicates).

### Data analysis

All results of the binding experiments (*K*_d_, *B*_max_, *K*_i_, *k*_off_, *k*_obs_) are given as the mean±standard error of the mean (SEM; at least three experiments). To determine the marker concentration in binding experiments, an individual calibration function was established for every binding experiment. They were generated by means of linear regression analysis with a 1/*x* ^2^ weighting in all cases. Based on the obtained calibration functions the bound marker concentrations were determined with Analyst v. 1.6.1 Software. Due to the dilution step of bound marker to 80 % after elution the concentrations have to be back calculated for final evaluation to 100 %. Specific binding was calculated as difference between total binding and nonspecific binding. Data from the binding experiment were analyzed by means of nonlinear regression analysis with Prism v. 6.07 (GraphPad Software). For saturation experiments the *One site*‐*specific binding* nonlinear regression tool was used to obtain saturation isotherms with calculated *K*_d_ (equilibrium dissociation constants) and *B*_max_ (maximum density of binding sites) values. For competition experiments the *One Site*‐*Fit* K_*i*_ nonlinear regression tool was used to obtain sigmoidal competition curves. The top level (total binding in absence of test compound) was set to 100 % and the bottom level (nonspecific binding) was set to 0 %. For competition curves with incomplete inhibition of Org25543 only the top level was set to 100 % and the bottom level was not constrained. The concentration, at which a test compound inhibited 50 % of specific marker binding (IC_50_ value), was transferred into *K*_i_ values (inhibition constant of the test compound) using the Cheng‐Prusoff equation. To obtain *k*_off_ (dissociation rate constants), *k*_obs_ (observed association rate constants) and dissociation t_1/2_ (half‐life) from kinetic experiments the nonlinear regression tools *Dissociation*‐*One phase exponential decay* and *One‐phase association* were used. Actual *k*_on_ (association rate constant) was calculated using following equation: *k*_on_=(*k*_obs_−*k*_off_)/L (with L being the used Org25543 concentration in the association experiment in [M]).

## Conflict of interest

The authors declare no conflict of interest.

## Supporting information

As a service to our authors and readers, this journal provides supporting information supplied by the authors. Such materials are peer reviewed and may be re‐organized for online delivery, but are not copy‐edited or typeset. Technical support issues arising from supporting information (other than missing files) should be addressed to the authors.

Supplementary
