# Supplementary material for: MS Binding Assays for Glycine Transporter 2 (GlyT2) Employing Org25543 as Reporter Ligand
Source: ChemMedChem. 2020 Sep 10;16(1):199–215. doi: 10.1002/cmdc.202000342 (PMC7821181; doi:10.1002/cmdc.202000342)
Supplement: Supplementary file 1 — Supplementary [file CMDC-16-199-s001.pdf]

ChemMedChem

Supporting Information

MS Binding Assays for Glycine Transporter 2 (GlyT2) Employing Org25543 as Reporter Ligand

Thomas M. Ackermann, Lars Allmendinger, Georg Höfner, and Klaus T. Wanner*

METHODS

Investigating filter binding of Org25543 on 96-well glass fiber filter plates

The investigation of filter binding of Org25543 was performed analog to *MS Binding Assay - general procedure* described in **Materials and methods**, except no GlyT1 membrane preparations were used for incubation. At first, the filters of 96-well glass fiber filter plates (AcroPrep Advance, glass fiber, 1.0 µm, 350 µL; Pall Corporation, Port Washington, NY, USA) were washed with water (3 x 200 µL) and methanol (3 x 200 µL) and subsequently incubated for 2 h at room temperature with different aqueous preincubation solutions (*m*/*m*): 200 µL of 0.5 % polyethylenimine (PEI), 1 % polyvinylpyrrolidone (PVP), 1 % Tween20, 0.5 % PVP-0.2 % Tween20 and 0.5 % L-lysine were used as preincubation solutions (triplicates), as control filters without incubation and pretreated filters with water were used (triplicates). During the incubation of the filters 50 nM Org25543 were incubated for 1 h in incubation buffer (10 mM HEPES, 120 mM NaCl, 2 mM KCl, 1 mM $MgCl_2$, 1 mM $CaCl_2$, pH 7.5) in polypropylene 96 well plates (1.2 mL well volume, Sarstedt, Nümbrecht, Germany) in a total volume of 250 µL at 37 °C in a shaking water bath. After incubation of filters and incubation samples, the preincubation solutions of the filters were removed by vacuum filtration and 210 µL aliquots of the incubation samples were transferred on the filters and were removed by vacuum filtration as well. The filters were washed with ammonium acetate buffer (3 x 200 µL, 154 mM, pH 7.4) and were dried at 50 °C for 1 h. After that the remaining Org25543 on the filters was washed down with acetonitrile (3 x 70 µL) and 52.5 µL ammonium bicarbonate buffer (5 mM, pH 7.8) was added to the samples to adjust the sample solvent to the mobile phase (5 mM ammonium bicarbonate buffer (pH 7.8)/acetonitrile (20:80, *v*/*v*)). Finally, the samples were subjected to LC-ESI-MS/MS quantification without further sample preparation. The peak areas of Org25543 were plotted against the different types of filter preincubations in a bar chart (Figure S3).

Investigating adsorption of Org25543 to polypropylene container material

To investigate the adsorption ability of Org25543 to polypropylene container material dilution series of Org25543 were prepared in reaction tubes (1.5 mL, PP, Sarstedt, Nümbrecht, Germany) using different solvents or solvent mixtures, respectively (H_2O, acetonitrile, H_2O/DMSO (80:20, *v/v*), H_2O/*N,N*-dimethylacetamide (DMA; 80:20, 90:10 and 95:5 *v/v*)). At least five concentration levels (10-fold concentration; 10 nM – 625 pM) were prepared in each solvent and these concentration levels were diluted again 1:10 (final concentrations of Org25543: 1 nM – 62.5 pM) in a mixture of ammonium bicarbonate buffer (5 mM, pH7.8) and acetonitrile containing 111 pM [$^2H_7$]Org25543 (final concentration: 100 pM) that the final sample solvent equals the mobile phase. Then the samples were subjected to LC-ESI-MS/MS quantification and the obtained peak area ratios of Org25543 and [$^2H_7$]Org25543 were plotted against the concentration of Org25543. By using linear regression analysis calibration curves were obtained for all dilution series and were compared to each other. The calibration curves are depicted in Figure S4.

Investigating the influence of *N,N*-dimethylacetamide (DMA) on the binding of Org25543 towards GlyT2 in competition experiments

This experiment was performed in the same way as it is described in the **Material and methods** part (see *4.13. Competition experiments)* except that Org25543 was stored in 100 % H_2O working solutions instead of 10 % aqueous DMA working solutions and that DMA was used as competitor in seven different concentrations (the concentration range of DMA goes from 6.0 % - 0.82 % which equals 647 mM – 888 µM (100 % DMA ≙ 10.79 M DMA)). The results are depicted in Figure S5.

Investigating the influence of 4-benzyloxy-3,5-dimethoxy-N-[1-[(dimethylaminocyclohexyl)methyl]benzamide (8) on the filter binding of Org25543

The investigation of the influence of **8** (see Figure 1) on the filter binding of Org25543 was performed analog to *MS Binding Assay - general procedure* described in **Materials and methods**, except no GlyT1 membrane preparations were used for incubation. At first, the filters of 96-well glass fiber filter plates (AcroPrep Advance,

glass fiber, 1.0 µm, 350 µL; Pall Corporation, Port Washington, NY, USA) were washed with water (3 x 200 µL) and methanol (3 x 200 µL) and subsequently pre-incubated for 2 h at room temperature with 0.5 % polyethylenimine (PEI). During the pre-incubation of the filters increasing concentrations of Org25543 (0.4 – 150 nM; triplicates) were incubated either in the absence of **8** or in the presence of three different concentrations of **8** (3, 30, 300 µM) for 1 h in incubation buffer (10 mM HEPES, 120 mM NaCl, 2 mM KCl, 1 mM $MgCl_2$, 1 mM $CaCl_2$, pH 7.5) in polypropylene 96 well plates (1.2 mL well volume, Sarstedt, Nümbrecht, Germany) in total volume of 250 µL at 37 °C in a shaking water bath. After the incubation times for the filters and incubation samples had been expired, PEI was removed from the filters by vacuum filtration and 210 µL aliquots of the incubation samples were transferred on the filters and were removed by vacuum filtration as well. The filters were washed with ammonium acetate buffer (3 x 200 µL, 154 mM, pH 7.4) and were dried at 50 °C for 1 h. After that the remaining Org25543 on the filters were washed down with acetonitrile (3 x 70 µL) containing [$^2H_7$]Org25543 (125 pM) and 52.5 µL ammonium bicarbonate buffer (5 mM, pH 7.8) was added to the samples to adjust the sample solvent to the mobile phase (5 mM ammonium bicarbonate buffer (pH 7.8)/acetonitrile (20:80, *v*/*v*)). Finally, the samples were subjected to LC-ESI-MS/MS quantification without further sample preparation. The bound Org25543 on the filters (in [pM]) were plotted against the nominal concentration of Org25543 in the incubation samples (in [nM]) (Figure S6).

Investigating the inhibition mode of glycine in preliminary Org25543 saturation experiments

In these preliminary experiments saturation experiments were performed in the same way as it is described in the **Material and methods** part except that Org25543 and GlyT2 were incubated either in the absence of glycine or in the presence of 2, 20 or 200 mM glycine. The saturation isotherms of a representative experiment are depicted in Figure S7. K_d and B_{max} value determined without glycine were normalized to 100 % (control) in all experiments and K_d and B_{max} values determined in the presence of the three glycine concentrations were seen in relation to the control values (in [%]). The results are compiled in Table S2.

GlyT2 MS Binding Assay – Competition experiment using glycine, DL-proline and *N*-oleoylglycine as test compound

These competition experiments were performed in the same way as it is described in the **Material and methods** part. The results are depicted in Figure S8.

RESULTS

Figure S1. Product ion scans for the $[M+H]^+$ parent ions of ALX1393 and Org25543 with the most intensive product ions.

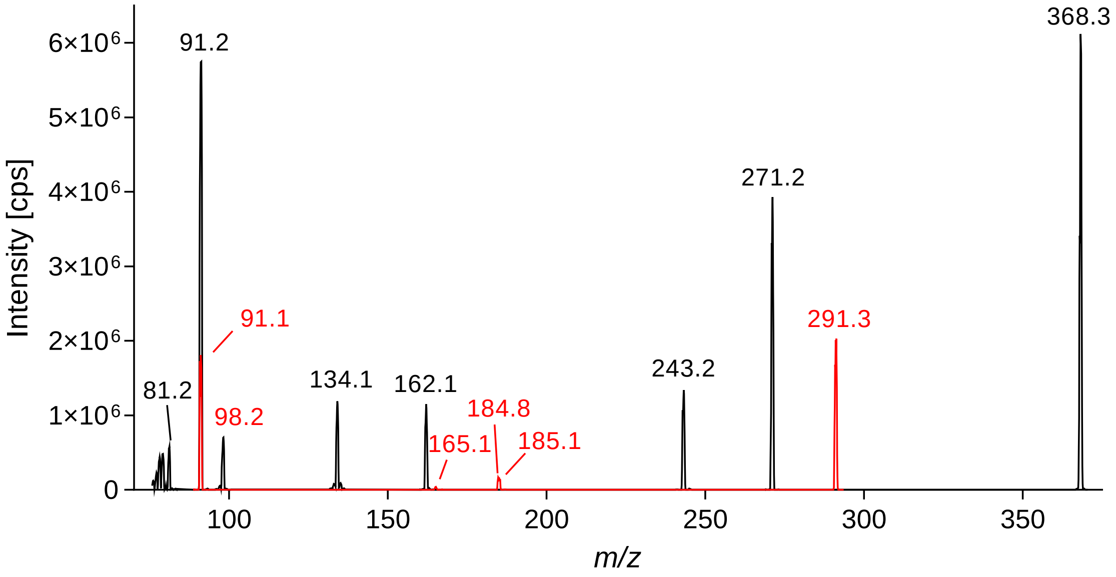

Depicted are the most intensive product ions of ALX1393 (m/z 396.1) in red and Org25543 (m/z 413.2) in black.

Figure S2. Representative LC-ESI-MS/MS chromatogram of ALX1393

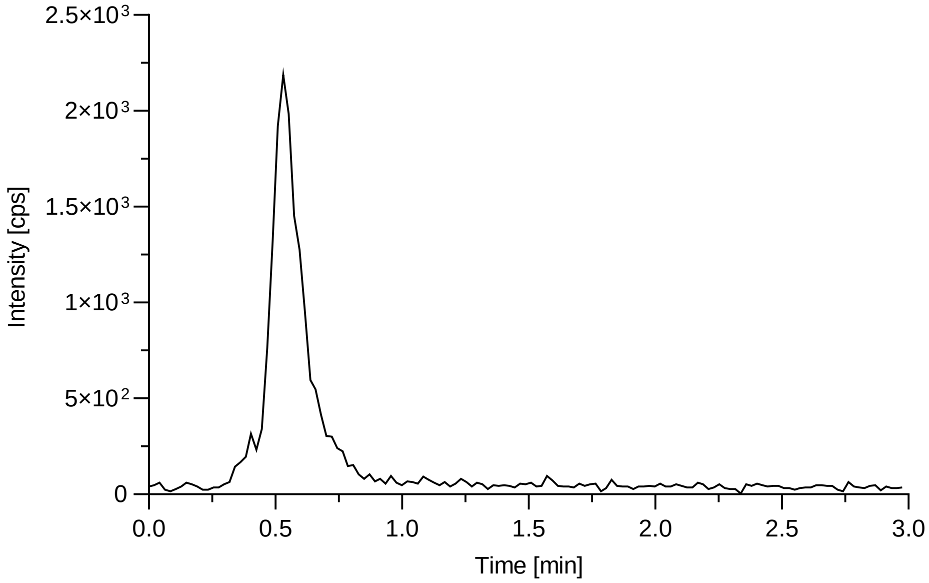

MRM chromatogram of 100 pM ALX1393 (*m*/*z* 396.1/291.0) diluted in ammonium bicarbonate buffer (5 mM, pH 7.8)/acetonitrile (40:60, *v*/*v*). For LC a Luna 3µ C8(2) (50 mm x 2 mm, 3 µm) column was used as stationary phase in combination with a mobile phase consisting of ammonium bicarbonate buffer (5 mM, pH 7.8)/acetonitrile (40:60, *v*/*v*) at a flow rate of 600 µL min^{-1}. Injection volume was set to 45 µL and temperature to 25 °C. A capacity factor *k* of 0.35 was achieved for ALX1393.

Figure S3. Investigating filter binding of Org25543 on 96-well glass fiber filter plates

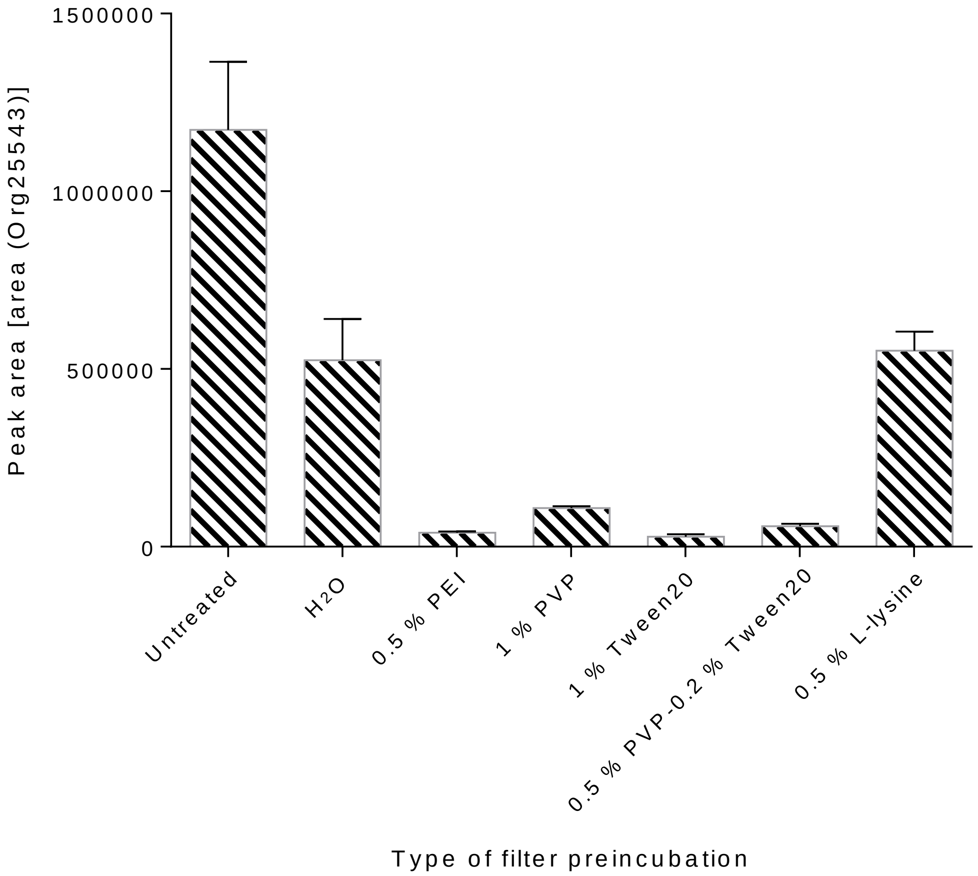

The results are given as mean ± standard deviation (n = 3). The preincubation solution with the smallest peak area shows to the lowest filter binding of Org25543. 0.5 % PEI and 1 % Tween20 were identified as the best solutions to reduce the filter binding of Org25543.

Figure S4. Investigating adsorption of Org25543 to polypropylene container material

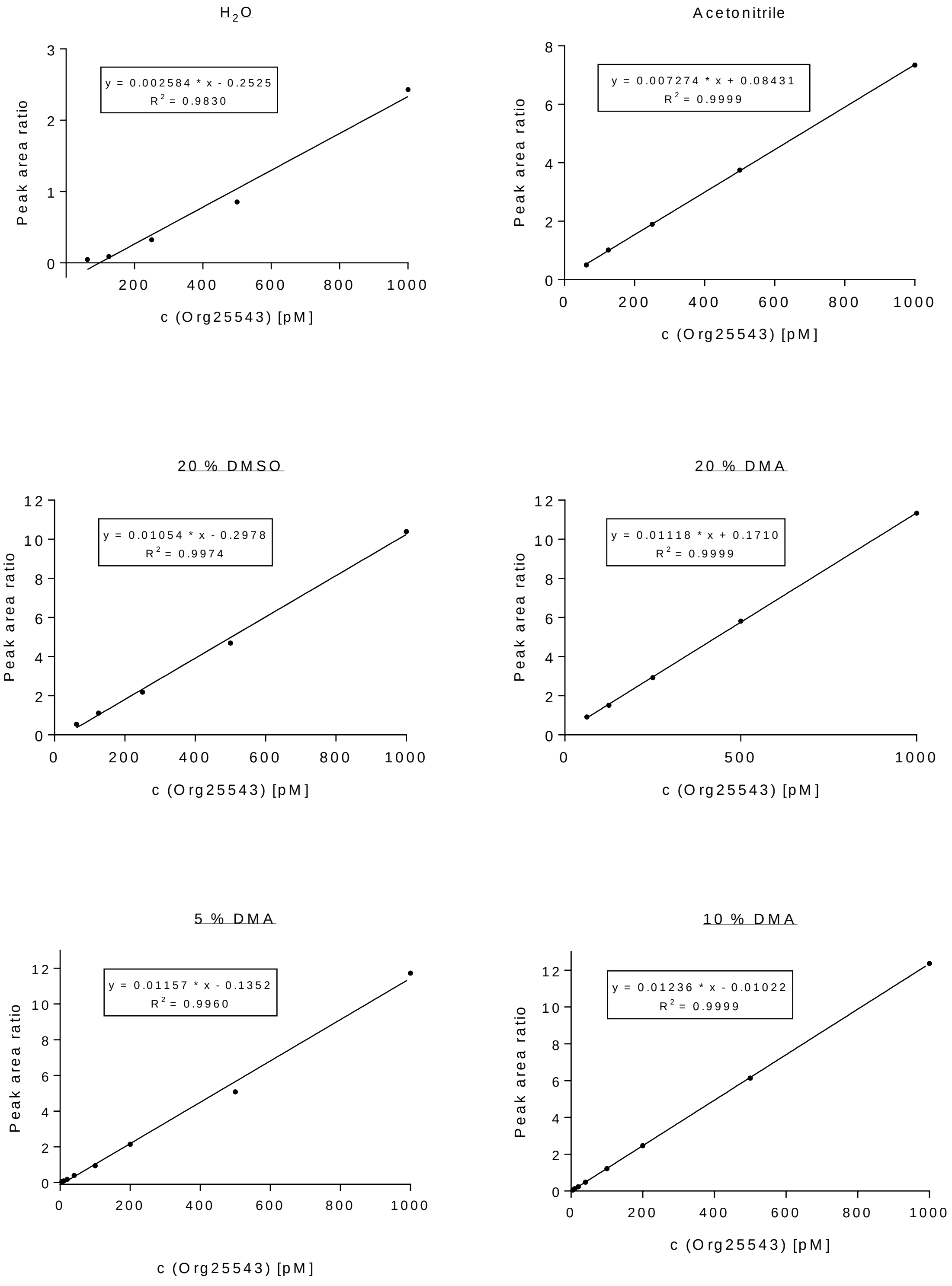

Figure S4 shows that the quality of calibration curves as well as the amount of measured Org25543 increases in the presence of an organic solvent. Since we were already experienced in our group with DMA as additive, we decided to continue with it.

As it can be seen in the bottom row a concentration of 5 % DMA in water already improves the quantification of Org25543 compared to pure water dilutions and 10 % DMA in water is enough to obtain satisfying calibration curves, so following solutions of Org25543 were all prepared in 10 % aqueous DMA.

Figure S5. Investigating the influence of *N,N*-dimethylacetamide (DMA) on the binding of Org25543 towards GlyT2 in competition experiments

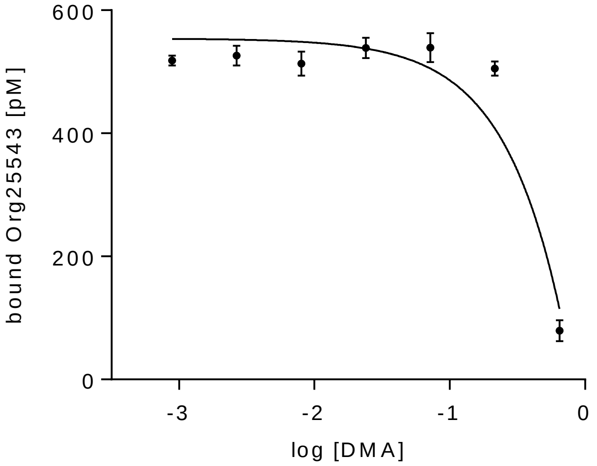

The results are given as mean ± standard deviation (n = 3). This competition experiment shows that DMA has no influence on the Org25543 binding at GlyT2 up to a concentration of 2 % (≙ 215 mM). Since the DMA concentration in binding samples was in all experiments 0.4 %, it should not have influenced the results of the performed experiments.

Figure S6. Investigating the influence of 4-benzyloxy-3,5-dimethoxy-N-[1-[(dimethylaminocyclohexyl)methyl]benzamide (8) on the filter binding of Org25543

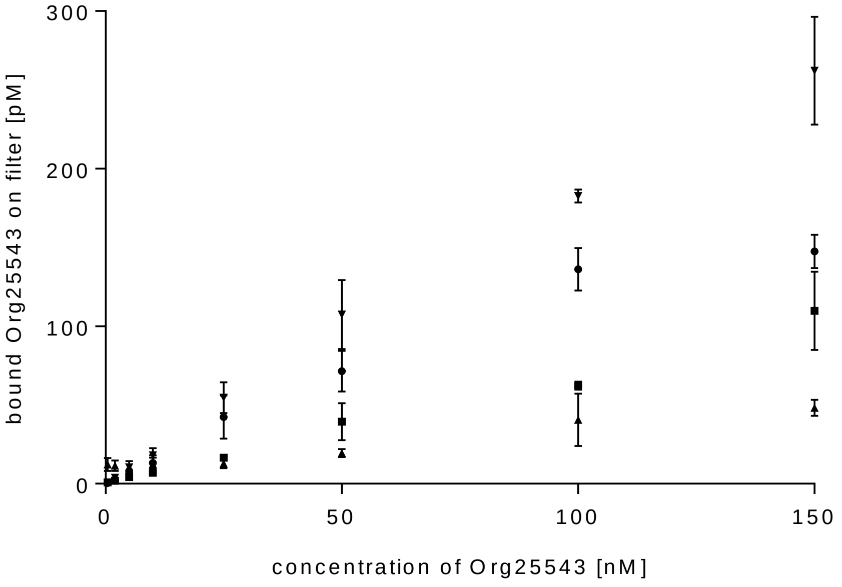

The results are given as mean ± standard deviation (n = 3). Figure S6 shows the filter binding of Org25543 in the absence of **8** (▾) and in the presence of 3 µM (•), 30 µM (▪) and 300 µM (▴) of **8**. As it can be seen the filter binding of Org25543 decreases in the presence of increasing concentrations of **8**, whereas in the absence of **8** the concentration of filter bound Org25543 was the highest. As filter binding is a part of the non-specific binding determined in binding experiments, the decreased filter binding is problematic especially for high concentrations in saturation experiments, in which the determined non-specific binding is lower due to the presence of **8** than the non-specific binding that is included in the total binding due to the absence of **8**. This means that specific binding still contains a small percentage of non-specific binding after subtracting the determined non-specific binding from total binding. However, the lower the Org25543 concentration gets, the more negligible is this effect. In competition experiments, for example, in which Org25543 is used in a concentration of 10 nM, the concentration of bound reporter ligand (total binding) lies between 350 and 400 pM, whereas the difference between non-specific binding of Org25543 determined with heat-shocked GlyT2 membrane fragments and non-specific binding determined with **8** lies between 5-8 pM in all cases we compared them to each other. This difference accounts just for 1-2 % of total binding which has hardly any effect on the result of a

competition experiment. But especially for saturation experiments, we decided to determine the non-specific binding without **8** as competitor and used heat-shocked GlyT2 membrane fragments, so that Org25543 is not able to bind to the target protein any more due to denaturation of it.

Figure S7. Investigating the inhibition mode of glycine in preliminary Org25543 saturation experiments

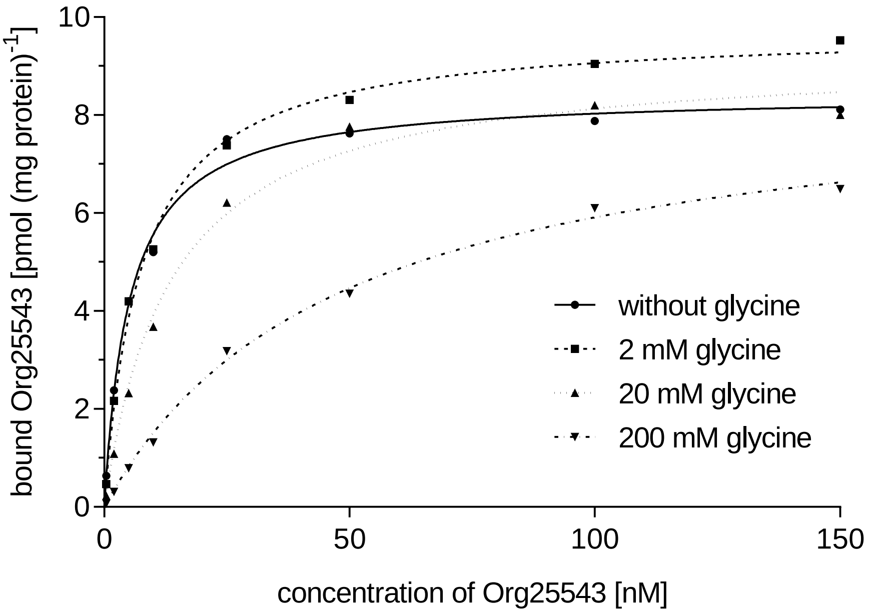

Representative preliminary Org25543 saturation experiment in absence of glycine and presence of three different glycine concentrations (2, 20, 200 mM). Depicted is the specific binding of every incubation condition resulting in four saturation isotherms which were determined by means of non-linear regression analysis. Without glycine a K_d = 5.18 nM and a B_{max} = 8.45 pmol (mg protein)$^{-1}$, for the incubation in the presence of 2 mM glycine a K_d = 7.56 nM and a B_{max} = 9.75 pmol (mg protein)$^{-1}$, for the incubation in the presence of 20 mM glycine a K_d = 13.55 nM and a B_{max} = 9.24 pmol (mg protein)$^{-1}$ and for the incubation in the presence of 200 mM glycine a K_d = 47.84 nM and a B_{max} = 8.74 pmol (mg protein)$^{-1}$ were found. These results indicate increasing K_d values of Org25543 for GlyT2 when it is incubated with increasing concentrations of glycine whereas B_{max} stays nearly the same.

Figure S8. GlyT2 MS Binding Assay – Competition experiment using glycine, DL-proline and *N*-oleoylglycine as test compound

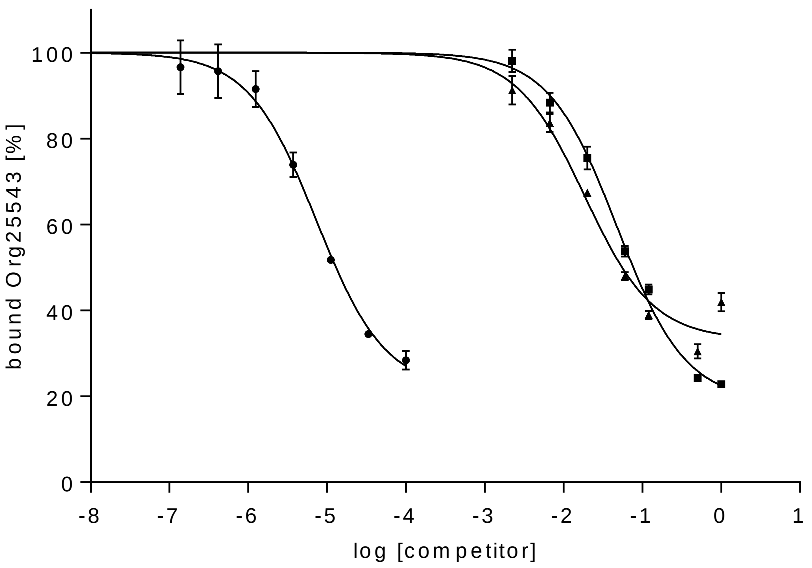

The results are given as mean ± standard deviation (n = 3). In all three competition curves it can be seen that the competitors glycine (▲), DL-proline (▪) and *N*-oleoylglycine (•) are not able to displace Org25543 completely from GlyT2 up to the highest concentration investigated, so the curves do not reach the non-specific binding level (0 %).

Table S1. Validation Results of LC-ESI-MS/MS method for Org25543

intra-batch accuracy and precision															
**Samples (n)**	**Series 1**			**Series 2**			**Series 3**			**Series 4**			**Series 5**		
	**M**	**A**	**P**	**M**	**A**	**P**	**M**	**A**	**P**	**M**	**A**	**P**	**M**	**A**	**P**
**Kal 5 pM (6)**	5.054	101.1	1.7	5.078	101.6	3.3	5.102	102.0	2.6	4.929	98.6	3.5	5.027	100.5	5.8
**Kal 10 pM (3)**	9.789	97.9	1.0	9.594	95.9	4.0	9.421	94.2	0.5	10.62	106.2	4.3	10.54	105.4	4.1
**Kal 20 pM (3)**	19.69	98.5	1.5	19.48	97.4	2.1	19.40	97.0	1.8	19.98	99.9	1.1	21.30	106.5	2.0
**Kal 40 pM (3)**	38.78	96.9	1.0	39.01	97.5	0.6	38.65	96.6	3.3	39.71	99.3	0.7	42.64	106.6	2.2
**Kal 100 pM (3)**	99.20	99.2	0.5	99.13	99.1	0.5	97.60	97.6	1.4	99.70	99.7	2.2	105.2	105.2	2.5
**Kal 200 pM (3)**	197.9	99.0	0.5	194.8	97.4	1.9	204.9	102.4	1.1	195.5	97.7	1.6	211.1	105.5	1.8
**Kal 500 pM (3)**	511.5	102.3	0.9	521.2	104.2	0.9	523.2	104.6	0.4	500.6	100.1	1.3	532.7	106.5	3.0
**Kal 1000 pM (3)**	1043	104.3	1.6	1055	105.5	1.3	1035	103.5	1.3	1001	100.1	0.8	1086	108.6	1.6
**Equation of calibration***	y = 1.212 x + -0.00898 (r = 0.9990)			y = 1.150 x + -0.00333 (r = 0.9976)			y = 1.153 x + -0.00196 (r = 0.9975)			y = 1.218 x + 0.00562 (r = 0.9983)			y = 1.304 x + 0.00192 (r = 0.9983)		
**QC 15 pM (6)**	15.74	107.3	2.4	15.41	102.7	3.7	16.32	108.8	1.6	14.45	96.4	3.7	15.23	101.6	5.3
**QC 300 pM (6)**	304.0	104.4	0.9	306.4	102.1	0.9	323.4	107.8	0.7	300.7	100.2	1.2	324.1	108.0	0.9
**QC 800 pM (6)**	820.0	110.0	1.2	830.7	103.8	0.9	868.9	108.6	0.7	770.3	96.3	0.7	813.4	101.7	1.2

inter-batch accuracy and precision			
	**M**	**A**	**P**
**LLOQ 5 pM**	5.038	100.8	3.6
**QC 15 pM**	15.43	102.9	5.2
**QC 300 pM**	311.7	103.9	3.4
**QC 800 pM**	820.7	102.6	4.0

M Mean [pM]; **A** Accuracy [%]; **P** Precision [%]; * Weighting factor: $1/x^2$

Table S2. Investigating the inhibition mode of glycine in preliminary Org25543 saturation experiments

	Incubation conditions		
**Parameters**	**2 mM glycine**	**20 mM glycine**	**200 mM glycine**
K_d[a] [%]	129 ± 24	222 ± 56	814 ± 154
B_{max}[a] [%]	106 ± 13	101 ± 12	---[b]

[a]K_d and B_{max} are seen in relation to the control values determined without glycine which were normalized to 100 %. [b]B_{max} was not included due to Org25543 concentrations which are not high enough to determine reliable results. All results are presented as mean ± SD from two independently performed experiments.
